# Unraveling the Regulatory Mechanism of Color Diversity in *Camellia japonica* Petals by Integrative Transcriptome and Metabolome Analysis

**DOI:** 10.3389/fpls.2021.685136

**Published:** 2021-06-11

**Authors:** Mingyue Fu, Xu Yang, Jiarui Zheng, Ling Wang, Xiaoyan Yang, Yi Tu, Jiabao Ye, Weiwei Zhang, Yongling Liao, Shuiyuan Cheng, Feng Xu

**Affiliations:** ^1^College of Horticulture and Gardening, Yangtze University, Jingzhou, China; ^2^Department of Forestry Ecology, Hubei Ecology Polytechnic College, Wuhan, China; ^3^National R&D Center for Se-Rich Agricultural Products Processing, Wuhan Polytechnic University, Wuhan, China

**Keywords:** *Camellia japonica*, petal, anthocyanin, color diversity, transcriptome, transcription factor, structural genes, metabolome

## Abstract

*Camellia japonica* petals are colorful, rich in anthocyanins, and possess important ornamental, edible, and medicinal value. However, the regulatory mechanism of anthocyanin accumulation in *C. japonica* is still unclear. In this study, an integrative analysis of the metabolome and transcriptome was conducted in five *C. japonica* cultivars with different petal colors. Overall, a total of 187 flavonoids were identified (including 25 anthocyanins), and 11 anthocyanins were markedly differentially accumulated among these petals, contributing to the different petal colors in *C. japonica*. Moreover, cyanidin-3-*O-*(6^″^-*O-*malonyl) glucoside was confirmed as the main contributor to the red petal phenotype, while cyanidin-3-*O-*rutinoside, peonidin-3-*O-*glucoside, cyanidin-3-*O-*glucoside, and pelargonidin-3-*O-*glucoside were responsible for the deep coloration of the *C. japonica* petals. Furthermore, a total of 12,531 differentially expressed genes (DEGs) and overlapping DEGs (634 DEGs) were identified by RNA sequencing, and the correlation between the expression level of the DEGs and the anthocyanin content was explored. The candidate genes regulating anthocyanin accumulation in the *C. japonica* petals were identified and included 37 structural genes (especially *CjANS* and *Cj4CL*), 18 keys differentially expressed transcription factors (such as *GATA*, *MYB*, *bHLH*, *WRKY*, and *NAC*), and 16 other regulators (mainly including transporter proteins, zinc-finger proteins, and others). Our results provide new insights for elucidating the function of anthocyanins in *C. japonica* petal color expression.

## Introduction

As a visible trait of plants, color is of great significance to plant growth and development. Anthocyanins are water-soluble pigments belonging to the flavonoid family that mainly contribute to red and blue coloring. In recent years, a series of studies on anthocyanins has revealed their contribution to typical coloring in plants. For example, anthocyanins are involved in color formation in the skin of *Ziziphus jujuba* (Zhang Q. et al., 2020) and *Malus domestica* (Fang et al., 2019a, b). Anthocyanins are responsible for flower color and petal blotches in *Paeonia suffruticosa* (Gu et al., 2019), *Salvia miltiorrhiza* (Jiang et al., 2020), and *Pleione limprichtii* (Zhang et al., 2019). Anthocyanins also contribute to fruit color, such as in *Hylocereus spp.* (Fan et al., 2020) and *Morus atropurpurea* (Huang et al., 2020). Moreover, anthocyanin has various other functions in plants, including in the protection against abiotic stresses, resistance to pathogens, tolerance to environmental stresses, and pollinator attraction (Schaefer et al., 2004; Zhang et al., 2015). Anthocyanins are also used as food colorants (Shahid et al., 2013). Compared with other food colorants, anthocyanins have strong antioxidant and free radical-scavenging properties, and thus they are associated with a variety of health benefits, and foods rich in anthocyanins are popular among consumers (Alvarez-Suarez et al., 2014; Smeriglio et al., 2016). However, artificially synthetic anthocyanins are inevitably used, and these contain various metal ions that are harmful to human health (Carocho et al., 2014). Thus, natural anthocyanins have great market potential. Anthocyanin-rich plant extracts have been used as substitutes for synthetic pigments in a wide variety of products; for instance, in dairy products, including cheese, fermented milk, and milk (Kitts and Tomiuk, 2013; de Mejia et al., 2015; Pineda-Vadillo et al., 2017).

Anthocyanins are synthesized *via* the flavonoid pathway and are regulated by many structural genes, including *CHS*, *CHI*, *DFR*, *F3H*, *UFGT*, and *ANS* (Hichri et al., 2011). Moreover, some transcription factors (TFs) affect the synthesis of anthocyanins by binding to the promoter regions of structural genes, such as bZIP, MYB, bHLH, MADS-box, and WD (An et al., 2017; Lu et al., 2018; Jian et al., 2019). Among them, the regulatory function of the MBW (MYB-bHLH-WD) protein complex consisting of MYB, bHLH, and WD in anthocyanin synthesis is well stablished (Feng et al., 2020). Besides, some zinc-finger proteins play a key role in the accumulation of anthocyanins (Shi et al., 2018; Bai et al., 2019). Some external environmental factors can also induce the formation of anthocyanins, such as UV-B, temperature, and drought (An et al., 2019; Yang et al., 2020).

Common sources of natural anthocyanins mainly include red grape marc, black carrot, purple potato, and onion (Ersus Bilek et al., 2017; Mourtzinos et al., 2018), but these cannot meet the increasing demand for natural food pigments. Therefore, finding new extraction sources of natural anthocyanins is necessary. The flowers of *Camellia japonica*, as one of the top 10 national flowers in China, are well-known for their striking colors. The flower color of *C. japonica* is undoubtedly caused by the hyperaccumulation of anthocyanins, and thus this flower might constitute a new natural anthocyanin source (Pan et al., 2020). However, the anthocyanins in the petals of *C. japonica* have not been sufficiently identified or quantified. In this study, we detected and quantified the composition and content of anthocyanins and clarified the regulatory network of anthocyanin biosynthesis in *C. japonica* using an integrated metabolome and transcriptome analysis. This study identified the candidate genes regulating the mechanism of *C. japonica* petal coloration, thus providing a foundation for the metabolic engineering of anthocyanin biosynthesis in the petals of *C. japonica*.

## Materials and Methods

### Plant Materials

**FIGURE 1:**
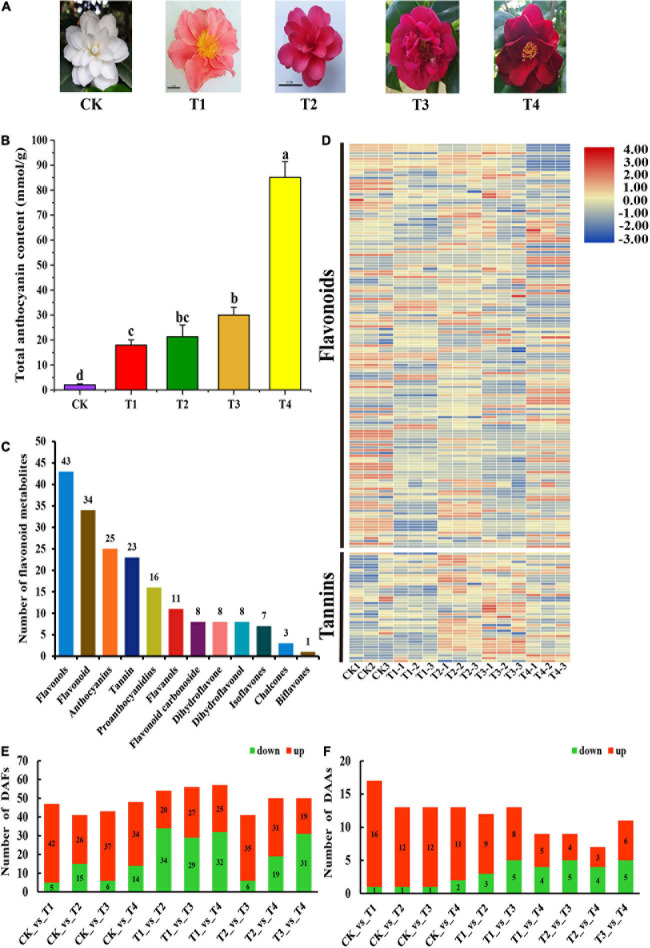
Phenotypes Comparison of phenotypes, composition and content of anthocyanins among five *C. japonica* petals. **(A)** Phenotypes of *C. japonica* petals. **(B)** Total anthocyanin content of *C. japonica* petals. All data were shown as mean ± SE (*n* = 3). Means with different letters at each treatment represented a significant difference at *p* ≤ 0.05. **(C)** Classification and statistical analysis of all metabolites detected. **(D)** The heatmap analysis of all metabolites relative content by TBtools. **(E)** Number of DAFs among all comparison units. **(F)** Number of DAAs among all comparison units.

Five *C. japonica* varieties were planted in Wunao Mountain National Forest Park (31°13′44^″^N, 114°59′17^″^E) in Macheng, Hubei Province, China. All collections were approved by the head of Wunao Mountain National Forest Park in Macheng. The petal colors of the five *C. japonica* varieties, namely “Niuxiaomeiyu” (CK), “Sishaluo” (T1), “Zaohongyang” (T2), “Dahongmudan” (T3), and “Huangjiatianerong” (T4) were white, pink, deep pink, red, and crimson, respectively (Figure 1A). Sixty petals were sampled and pooled from three individual plants, and three replicates were set for each *C. japonica* variety. Fresh petals were collected on April 30, 2020. All samples were sampled, immediately frozen in liquid nitrogen, and then stored at –80°C.

### Measurement of Total Anthocyanin Content

Approximately 0.1 g of *C. japonica* petal material was used to determine the total anthocyanin content as described by Mehrtens et al. (2005) with some modifications. Fresh petals were ground in 10 mL 95% ethanol (0.1 mol L^–1^ HCl) and extracted twice at 60°C for 1 h. The final volume of extraction solution was 25 mL (including ethanol-HCl), and the absorption at 520, 620, and 650 nm was measured using a sense microplate reader (435–301, HIDEX, Finland). The total anthocyanin content was calculated using the following formula: Q = Aλ × V × 1,000 / 489.72M (mmol/g FW), where Aλ = (A530–A620)–0.1 (A650–A620), V represents the volume of the extraction solution, and M represents the weight of the fresh sample. Ninety-five percent ethanol (0.1 mol L^–1^ HCl) was used as the blank control.

### Separation and Detection of Total Flavonoids in *Camellia japonica* Petals

Petal powder was obtained by crushing freeze-dried petals in a mixer mill (MM 400, Retsch). The extraction of total flavonoids was as follows: 100 mg powder was accurately weighed and extracted in 1.0 mL 70% aqueous methanol solution overnight at 4°C. The extract was centrifuged at a high speed, and the sediment was removed. The supernatant was absorbed using a solid-phase extraction cartridge (CNWBOND Carbon-GCB SPE Cartridge, 250 mg, 3 mL; ANPEL, Shanghai, China,^1^) and filtered with a nylon syringe filter (SCAA-104, 0.22 μm pore size; ANPEL, Shanghai, China,^2^) for liquid chromatography-mass spectrometry (LC-MS) analysis. The extraction solution was analyzed by an LC-electrospray ionization-tandem mass spectrometry (ESI-MS/MS) system (HPLC, Shim-pack UFLC SHIMADZU CBM30A system, Shimadzu, Japan,^3^; MS, 4500 Q TRAP, Applied Biosystems, United States,^4^). The HPLC and MS conditions were described by Chen J. et al. (2020).

### Identification and Quantitative Analysis of Metabolites

The metabolites eluted from the HPLC were monitored for each period by scheduled multiple reaction monitoring (MRM). The MRM signals were converted and analyzed using the software Analyst 1.6.3 (Metware Biotechnology Co., Ltd., Wuhan, China). Metabolite identification and quantification in this study were conducted following the commercially available standard Metabolites Database (Metware Biotechnology Co., Ltd., Wuhan, China) (Jiang et al., 2020) and public metabolite databases (Wishart et al., 2013; Zhu et al., 2013). The identified peak area of each compound was used for principal component analysis and orthogonal partial least squares-discriminant analysis. The differentially accumulated flavonoids (DAFs) and differentially accumulated anthocyanins (DAAs) were identified by R statistical software, and the screening criteria were as follows: metabolites with fold change ≥ 2 and fold changes ≤ 0.5 and variable importance in the project (VIP) score ≥ 1 were considered significantly differentially accumulated.

### RNA Extraction and Sequencing

All freeze-dried petals were ground on dry ice to extract the total RNA. After extraction with a Trizol reagent kit (Invitrogen, Carlsbad, CA, United States), the quality and integrity of the total RNA were assessed and checked using an Agilent 2100 Bioanalyzer (Agilent Technologies, Palo Alto, CA, United States) and RNase-free agarose gel electrophoresis, respectively. The mRNA was enriched by Oligo(dT) beads and fragmented into short fragments using fragmentation buffer. The short fragments were reversed transcribed into cDNA with random primers and synthesized to second-strand cDNA using DNA polymerase I, RNase, dNTP, and buffer. The QiaQuick PCR extraction kit (Qiagen, Venlo, Netherlands) was used to purify the cDNA fragments. Following end-repair and the addition of poly(A), and fragments were ligated to Illumina sequencing adapters. After selection by agarose gel electrophoresis and PCR amplification, all products were sequenced on the Illumina HiSeq2500 platform of Gene Denovo Biotechnology Co. (Guangzhou, China).

### RNA-seq Data Analysis and Annotation

To acquire high-quality clean reads for assembly and analysis, reads obtained from the sequencing platform were filtered by fastp (version 0.18.0) (Chen et al., 2018). To reduce the influence of the rRNA in the sample on the results, the software bowtie2 (Gene Denovo Biotechnology Co., Guangzhou, China) was used to align clean reads to the ribosome database (Langmead and Salzberg, 2012). The remaining unmapped reads were used for subsequent transcriptome analysis by removing the reads that could be mapped to the ribosomes. The clean reads were mapped to the reference genome (Xia et al., 2019; Tea Plant Information Archive,^5^) using HISAT2. 2.4 (Kim et al., 2015). The mapped reads of the five groups of samples were assembled by StringTie v1.3.1, and the fragments per kilobase of transcript per million mapped reads (FPKM) value was counted to quantify the expression (Pertea et al., 2015, 2016). All transcripts were annotated from databases, including the Gene Ontology (GO) database, Kyoto Encyclopedia of Genes and Genomes (KEGG) database, NCBI non-redundant (Nr) database, Swiss-Prot protein database, and Pfam database. The genes featuring a false discovery rate (FDR) below 0.05 and absolute fold change ≥ 2 and fold change ≤ 0.5 were considered as differentially expressed genes (DEGs). The DEGs among the five group samples were identified by DESeq2 for subsequent analysis.

### Weighted Gene Co-expression Network Analysis

The overlapping DEGs and DAAs were selected for co-expression network analysis *via* weighted gene co-expression network analysis (WGCNA) tools from the BMKCloud platform^6^. The content of overlapping DAAs was used as a trait for the WGCNA, and modules were obtained through WGCNA analysis with default settings. Furthermore, the correlation coefficients between the hub genes in the module and the DAAs were calculated using the OmicShare tools (Gene Denovo Co., Ltd., Guangzhou, China,^7^). The hub genes with DAAs Pearson’s correlation coefficient (PCC) values ≥ 0.95 or ≤ −0.9 were selected to draw the directed interaction network diagram.

### Transcription Factor Analysis

Considering the important role of TFs in anthocyanin synthesis, the TFs expressed in all samples were identified. All putative TFs were retrieved by searching against the transcription annotation file with the keyword “transcription factor” and the numbers and types of TFs were counted. Moreover, the expression levels of all TFs in the sample were analyzed by TBtools (Chen C. et al., 2020), and the PCC between these differentially expressed TFs and total anthocyanin content of samples was calculated. The TFs with | PCC| ≥ 0.9 were selected for subsequent analysis.

### Validation RNA-seq by Quantitative Real Time-PCR

Total RNA isolation was conducted by using an RNAprep Pure Plant Plus Kit (DP441, Tiangen, Beijing, China,^8^). First-strand cDNA was synthesized with HiScript III RT SuperMix for qPCR (+gDNA wiper) (R323, Vazyme, Nanjing, China,^9^) and the extracted RNA was used as the template. The LineGene 9600 Plus Fluorescent Quantitative PCR System (Bioer, Hangzhou, China) was used to perform quantitative real time (qRT)-PCR and AceQ Universal SYBR qPCR Master Mix (Q511, Vazyme) was selected as the fluorochrome. The primers for qRT-PCR were designed using the Integrated DNA Technologies tool^10^ and are listed in Table S1. To validate the results of the RNA-seq, 33 DEGs were randomly selected for qRT-PCR, and the *18S* gene was used as an internal control (Sun et al., 2014). The results of the qRT-PCR were calculated according to the 2^–Δ^ ^Δ^ ^*Ct*^ comparative Ct method (Schmittgen and Livak, 2008).

### Statistical Analysis

All raw RNA-seq data in the present study were uploaded to the Genome Sequence Archive in the BIG Data Center, Beijing Institute of Genomics (BIG), Chinese Academy of Sciences. The accession number is CRA003840, and the data are publicly accessible at https://bigd.big.ac.cn/gsa. Significant differences were calculated by SPSS 22.0 (SPSS Inc., Chicago, IL, United States) software for a one-way ANOVA analysis with a Turkey test, and significance was assessed at *P* ≤ 0.05. All data in this paper were expressed as means ± standard deviations (SD).

## Results

### Metabolic Differences Among the Petals of *Camellia japonica* Cultivar

To detect the metabolic mechanism of the color phenotype in the *C. japonica* petals, the total anthocyanins in the petals were measured. The total anthocyanin content of the T4 sample was 85.09 mM g^–1^, which was significantly higher than that of the other samples (Figure 1B). This was followed by the T1, T2, and T3 samples, the total anthocyanin contents of which were 17.96, 21.32, and 29.98 mmol g^–1^, respectively. The total anthocyanin content of the petals of the CK cultivar was significantly lower than the other varieties, with an anthocyanin content of 2.02 mmol g^–1^ (Figure 1B). These results corresponded with the color intensity of the *C. japonica* petals. Anthocyanins are a class of varied flavonoids with similar structural units. To further analyze the differences in flavonoid metabolites in the *C. japonica* petals of different colors, the anthocyanin and flavonoid metabolites were detected by UHPLC-ESI-MS/MS. A total of 187 flavonoids were identified from these five sample groups, and the relative content of all 187 flavonoids was analyzed (Figure 1D). The annotation, relative content, and classification of the 187 flavonoids are detailed in Supplementary Table 2. These flavonoids were classified into 12 categories, including flavonols, flavonoid, anthocyanins, tannin, proanthocyanidins, flavonols, flavonoid carbonoside, dihydroflavone, dihydroflavonol, isoflavones, chalcones, and biflavones. These categories contained 1 to 43 types of metabolites (Figure 1C).

Based on the data obtained from the metabolome, 47, 41, 43, 48, 54, 56, 57, 41, 50, and 50 types of DAFs were identified between the CK vs T1, CK vs T2, CK vs T3, CK vs T4, T1 vs T2, T1 vs T3, T1 vs T4, T2 vs T3, T2 vs T4, and T3 vs T4 groups, respectively (Figure 1E). Subsequently, the DAAs among these samples were obtained by analyzing the relative anthocyanin quantification results (Figure 1F). The results indicated that there were 16, 12, 12, and 11 types of anthocyanins that were up-regulated in T1, T2, T3, and T4, respectively, compared with CK, and these might be the key metabolites that influencing petal coloration in *C. japonica* (Figure 1F).

**FIGURE 2:**
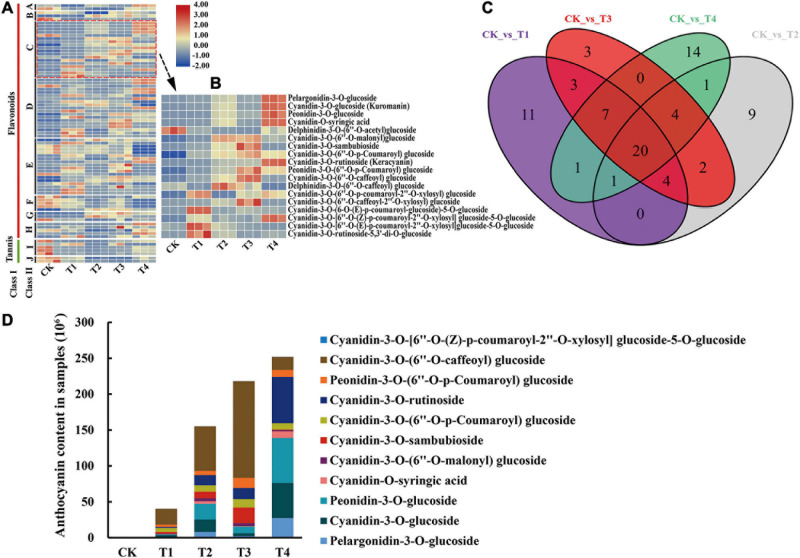
Differentially accumulated metabolites analysis of metabolome. **(A)** The heatmap analysis of all differentially accumulated metabolites by TBtools. A, chalcones; B, dihydroflavonol; C, anthocyanins; D, flavonoid; E, flavonols; F, flavonoid carbonoside; G, flavanols; H, isoflavones; I, tannin; J, proanthocyanidins. **(B)** The heatmap analysis of all DAAs according to the relative content in samples. **(C)** Venn analysis among CK vs T1, CK vs T2, CK vs T3, and CK vs T4. **(D)** The relative content of overlapping DAAs (11 anthocyanins) in five *C. japonica* cultivar petals.

A total of 80 DAFs were identified among all samples and the relative contents of these DAFs were illustrated in a heatmap (Figure 2A). Among these DAFs, 18 DAAs were included, and their relative contents are detailed in Figure 2B. Moreover, the overlapping metabolites were identified. The results showed that 20 DAFs were shared among CK vs T1, CK vs T2, CK vs T3, and CK vs T4 (Figure 2C). These 20 metabolites included five flavonoids, three flavonols, one dihydroflavonols, and 11 of anthocyanins. The classification results of these 11 anthocyanins indicated that most were cyanidin glucosides and a small amount were pelargonidin glucosides and peonidin glucosides (Figure 2D).

### Overview of the Transcriptome Data

To further study the molecular mechanism of *C. japonica* petal coloring, RNA-seq analysis was performed. Fifteen libraries were established using the five varieties of samples (three biological replicates for each variety), and a total of 39.19 to 58.82 Gb raw reads for each sample were obtained by sequencing. Clean reads were obtained after filtering low-quality reads and accounted for 99.72–99.83% of the raw data (Supplementary Table 3). The Q20 and Q30 values of each library were greater than or equal to 96.72 and 91.43%, respectively (Supplementary Table 4). The GC contents of each sample ranged from 46.21–47.73% (Supplementary Table 4). HISAT2. 2.4 was used to map clean reads to the reference genome, and the mapping ratio ranged between 73.40 and 76.89% (Supplementary Table 5). In addition, more than 86.77% of the reads were mapped to the exon region of the reference genome (Supplementary Table 6). Ultimately, the sequence and expression information of 57,410 genes was obtained for subsequent analysis. Heatmap analysis of the samples based on the FPKM values showed that all the biological replicates exhibited similar expression patterns, indicating the high reliability of our sequencing data (Supplementary Figure 1A). All these data proved that the sequencing quality was sufficient for further analysis.

### Identification of DEGs in *Camellia japonica* Petals

A total of 12,531 DEGs were characterized among all samples by analyzing the FPKM value of transcripts obtained from the transcriptome data. In detail, there were 4,984, 4,374, 6,139, 5,969, 5,475, 4,371, 6,784, 5,894, 7,337, and 8,308 DEGs, respectively in the 10 comparison groups, including CK vs T1, CK vs T2, CK vs T3, CK vs T4, T1 vs T2, T1 vs T3, T1 vs T4, T2 vs T3, T2 vs T4, and T3 vs T4 (Figure 2C). Among these comparison groups, the T3 vs T4 group had the largest number of up-regulated and down-regulated DEGs (Figure 2C). Furthermore, the overlap DEGs among the CK vs T1, CK vs T2, CK vs T3, and CK vs T4 groups were screened using the venn diagram function in TBtools (Chen C. et al., 2020). The results indicated that 634 genes were differentially expressed in these groups, which indicated that these DEGs might have key functions in the color expression of different petals (Supplementary Figure 1B).

### Functional Annotation of DEGs

**FIGURE 3:**
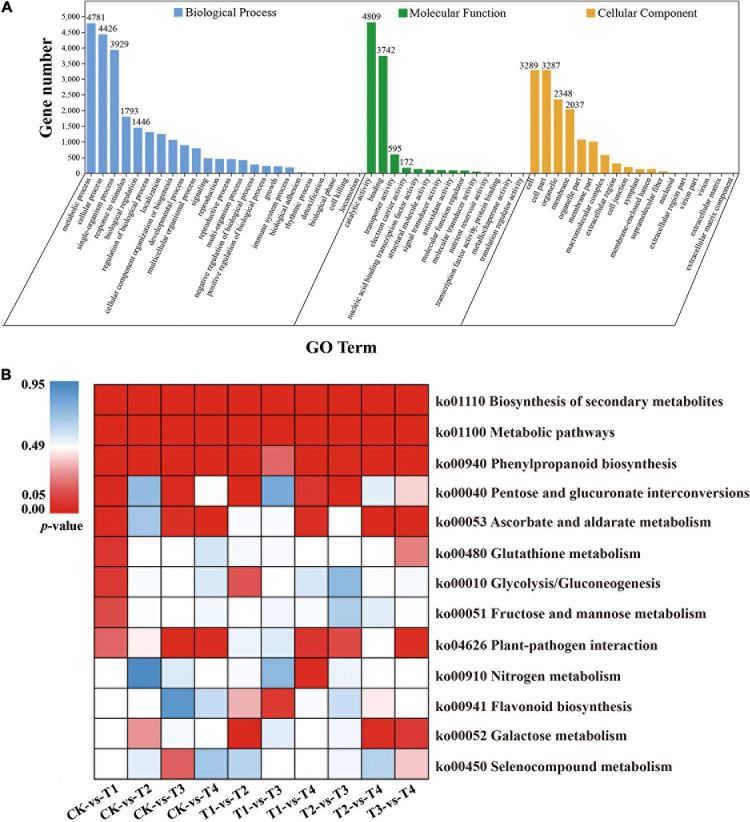
GO and KEGG enrichment analysis of all DEGs. **(A)** GO enrichment results of all DEGs **(B)** Enrichment of the top 13 KEGG pathways of all DEGs according to the *p*-value. The red represents significant enrichment.

All DEGs were mapped to the GO database and further classified into three classifications, including cellular component, molecular function, and biological process. In the cellular component category, more than 71.2% of DEGs were enriched in cell, cell part, organelle, and membrane terms (Figure 3A). In the biological process category, most of the DEGs were mapped to metabolic process (4,781, 38.15%), cellular process (4,426, 35.3%), and single-organism process (3,929, 31.4%) terms (Figure 3A), but for molecular function, nearly 69.36% of DEGs were mapped to catalytic activity and binding terms (Figure 3A). For the overlapping DEGs (634 DEGs), the GO annotation has the similar results (Supplementary Figure 2A). For the KEGG annotation results, 12,531 DEGs among all samples were mapped to 129 KEGG pathways. Among these pathways, biosynthesis of secondary metabolites, metabolic, and phenylpropanoid biosynthesis pathways were significantly enriched (Figure 3B). The KEGG annotation results of the overlapping DEGs (634 DEGs) showed that most enrichment pathway were photosynthesis-antenna proteins, ascorbate and aldarate metabolism, zeatin biosynthesis and protein export (Supplementary Figure 2B).

### The Key DEGs Responsible for the Anthocyanin Biosynthesis Pathway

**FIGURE 4:**
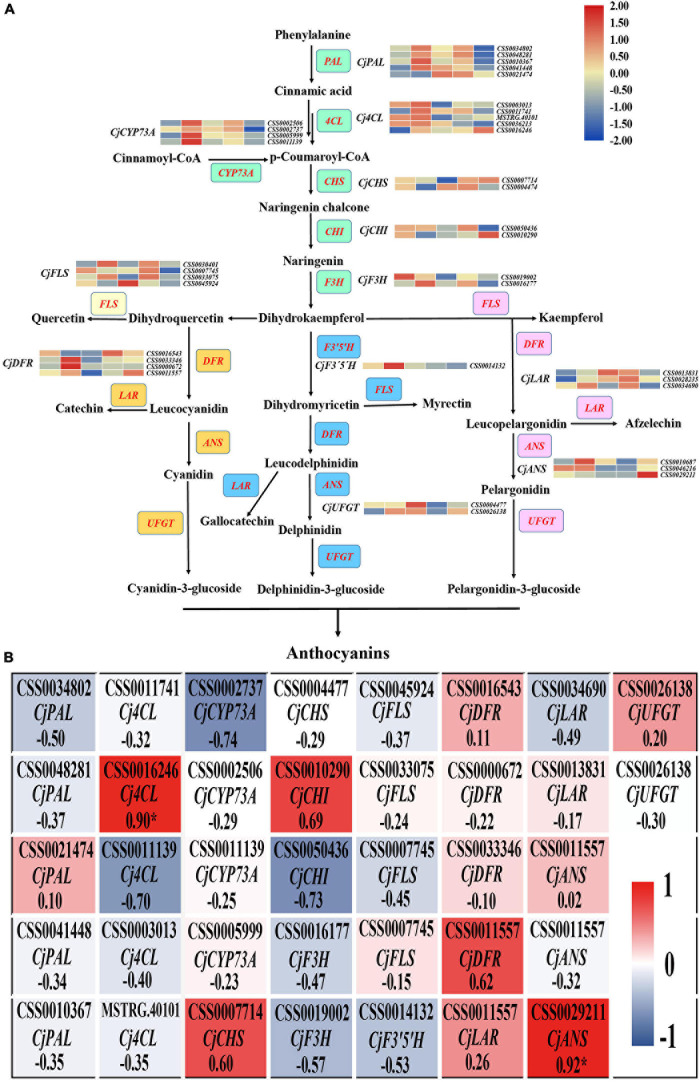
The analysis of DEGs in anthocyanin biosynthesis pathway. **(A)** The identification of all DEGs in anthocyanin biosynthesis pathway. The red font represents DEGs and the colored boxes represent the different branches of anthocyanin synthesis. **(B)** The heatmap analysis of all DEGs in anthocyanin biosynthesis pathway according to the FPKM value.

To explore the difference in anthocyanin biosynthesis among the petals from the five cultivars, DEGs in the anthocyanin synthesis pathway were identified, including the phenylpropanoid biosynthesis, flavonoid biosynthesis, and anthocyanin biosynthesis pathways. The results revealed that there were 37 DEGs enriched in the anthocyanin synthesis pathway, including *CjPAL*, *Cj4CL*, *CjCYP73A*, *CjCHS*, *CjCHI*, *CjF3H*, *CjFLS*, *CjF3′5′H*, *CjDFR*, *CjLAR*, and *CjANS* (Figure 4A). The PCC between the expression level of these DEGs and the total anthocyanin content was further calculated. The results showed that 10 DEGs positively regulated the anthocyanin synthesis, whereas 27 DEGs negatively regulated anthocyanin synthesis. Among these DEGs, the expression level of two DEGs, namely *CjANS* (*CSS0029211*) and *Cj4CL* (*CSS0016246*), indicated a significant positive correlation with the total anthocyanin content in the samples (PCC > 0.9, Figure 4B), suggesting that these two DEGs may have an essential role in anthocyanin accumulation.

### Identification TFs Related to Anthocyanin Biosynthesis

Transcription factors are important regulators of anthocyanin accumulation and they typically act by controlling the expression of structural genes in the anthocyanin biosynthesis pathway. In this study, a total of 883 TFs were identified by searching the transcriptome annotation results. The classified results indicated that most of these TFs belonged to the *AP2/ERF*, *bHLH*, *MYB*, *WRKY*, *GATA*, *NAC*, and *bZIP* family (Supplementary Figure 3A). Furthermore, the differentially expressed TFs were characterized by analyzing their FPKM values (Supplementary Figure 3A). By calculating the PCC between the expression level of these TFs and the total anthocyanins content, 19 key TFs (| PCC| ≥ 0.9) involved in the accumulation of anthocyanins were identified, including six negative regulators and 13 positive regulators. These six negative regulators, including one *MYB*, two *bHLHs*, one *WRKY*, one *TCP*, and one *AP2/ERF* gene, likely act as repressors in anthocyanin synthesis. However, the 13 positive regulators, including one *GATA*, two *MYBs*, three *bHLHs*, two *TCPs*, three *AP2/ERFs*, one *RAV*, and one *NAC* gene, might act as promotors of anthocyanin accumulation (Table 1). Furthermore, the expression level of these key TFs was illustrated in a heatmap, and the results showed that the positive regulators exhibited the highest expression level in T4, whereas the negative regulators exhibited the highest expression level in T1 (Supplementary Figure 3B).

**TABLE 1:** The candidate TFs involved in anthocyanin accumulation.

**Transcription factor**	**Gene ID**	**Annotation**	**Correlation with total anthocyanin**	***p*-value**
GATA	CSS0049672	GATA transcription factor 1	0.93	0.0212
MYB	CSS0015300	MYB family transcription factor	–0.97	0.0073
	CSS0014780	transcription factor R2R3-MYB1	0.93	0.0206
	CSS0045979	PREDICTED: transcription factor MYB86	0.93	0.0209
bHLH	MSTRG.36794	transcription factor bHLH51-like	–0.96	0.0110
	CSS0025568	PREDICTED: transcription factor bHLH106-like	–0.94	0.0157
	CSS0019162	PREDICTED: transcription factor bHLH137	0.97	0.0060
	CSS0029144	PREDICTED: transcription factor bHLH71	0.97	0.0071
	CSS0018697	PREDICTED: transcription factor bHLH11	0.95	0.0121
WRKY	CSS0016070	PREDICTED: WRKY transcription factor 44	–0.95	0.0124
TCP	CSS0042452	PREDICTED: transcription factor TCP20-like	–0.90	0.0353
	CSS0010873	PREDICTED: transcription factor TCP23-like	0.96	0.0094
	MSTRG.27463	transcription factor TCP4-like	0.95	0.0142
AP2/ERF	CSS0018330	transcription factor APETALA2	–0.92	0.0256
	CSS0002584	PREDICTED: ethylene-responsive transcription factor ERF023	0.98	0.0030
	CSS0009504	PREDICTED: ethylene-responsive transcription factor ERF014-like	0.98	0.0046
	CSS0048980	PREDICTED: AP2/ERF and B3 domain-containing transcription factor At1g50680-like isoform X2	0.95	0.0141
RAV	CSS0037887	RAV transcription factor	0.96	0.0090
NAC	CSS0036947	NAC transcription factor 037	0.99	0.0011

### Identification of Anthocyanin Synthesis-Related DEGs by WGCNA

**FIGURE 5:**
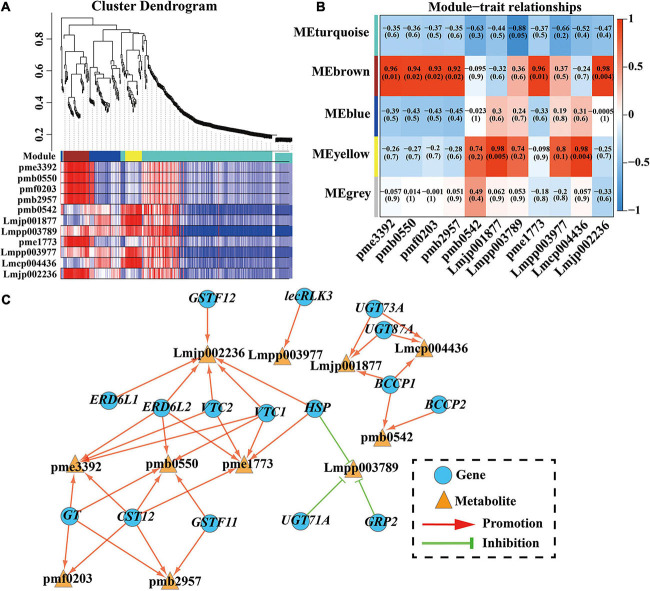
WGCNA results of 634 DEGs (with 11 DAAs PCC ≥ 0.95 or ≤ –0.90). **(A)** Hierarchical clustering tree (cluster dendrogram) results showed 5 expression modules, labeled with different colors. **(B)** Module-anthocyanin relationship analysis. The value inside each box represents Pearson’s correlation coefficient between the module with anthocyanin, and the number in each parentheses represents *p*-value. The color scale on the right represents the degree of correlation between modules and anthocyanins and the red represent high correlation. **(C)** The directed interaction network diagram between DEGs and anthocyanin. Red line represents the promotion of synthesis, while green “T” represents inhibition of accumulation. Blue circles represent DEGs and orange triangles represent anthocyanin. pme3392, pelargonidin-3-*O-*glucoside; pmb0550, cyanidin-3-*O-*glucoside; pmf0203, peonidin-3-*O-*glucoside; pmb2957, cyanidin-*O-*syringic acid; pme1773, cyanidin-3-*O-*(6^″^-*O-*malonyl) glucoside; Lmjp002236, cyanidin-3-*O-*sambubioside; Lmpp003789, cyanidin-3-*O-*(6^″^-*O-*p-Coumaroyl) glucoside; pmb0542, cyanidin-3-*O-*rutinoside; Lmjp001877, peonidin-3-*O-*(6^″^-*O-*p-Coumaroyl) glucoside; Lmpp003977, cyanidin-3-*O-*(6^″^-*O-*caffeoyl) glucoside; Lmcp004436, cyanidin-3-*O-*[6^″^-*O-*(Z)-p-coumaroyl-2^″^-*O-*xylosyl] glucoside-5-*O-*glucoside.

To investigate the gene regulatory network of anthocyanin accumulation in the *C. japonica* petals, WGCNA was conducted using the FPKM values of the overlapping DEGs (634 DEGs) and DAAs (11 anthocyanins) as source data. Five modules were identified in the cluster dendrogram, donated as turquoise, brown, blue, yellow, and gray modules (Figure 5A). Analysis of the module-anthocyanin relationships revealed that the brown, turquoise, and yellow modules have high PCCs with the content of anthocyanins (Figure 5B). To explore the expression patterns of the genes in these modules, heatmaps were conducted using the FPKM value of the genes in these modules. The heatmap results of the brown module proposed that the co-expressed genes had a higher expression level only in T3, showing significant sample specificity (Supplementary Figure 4A). In the turquoise module, the co-expressed genes were highly expressed in CK (Supplementary Figure 4B), whereas in the yellow module, the co-expressed genes had the highest expression level in T3 and the lowest expression level in CK (Supplementary Figure 4C). Moreover, a directed interaction network diagram was constructed using the PCCs between the hub genes in the brown, turquoise, and yellow module (PCC ≥ 0.95 or PCC ≤ −0.9) and the content of the 11 anthocyanins. As shown in Figure 5C, 16 genes in the modules were selected to construct the regulatory network, including two *CjGSTFs*, three *CjUGTs*, *CjGT*, two *CjERD6Ls*, *CjGRP2*, two *CjVTCs*, *CjHSP*, two *CjBCCPs*, *CjlecRLK3*, and *CjCST12*. All the anthocyanins were positively regulated by these genes, except for cyanidin-3-*O-*(6^″^-*O-*p-Coumaroyl) glucoside. The above results indicate that these 16 genes may play essential roles in anthocyanin synthesis in *C. japonica*.

### Verification of the Results in RNA-seq by qRT-PCR

To validate the accuracy of the RNA-seq data, 33 genes were randomly selected for qRT-PCR. The relative expression levels of these 33 genes were normalized to the expression of *18S* rRNA (Supplementary Figure 5). Further analysis revealed that the expression levels of these genes were significantly correlated with the RNA-seq results (Supplementary Figure 6, R^2^ > 0.75), indicating that the RNA-seq data were credible and accurate.

## Discussion

### Anthocyanin Identification in the *Camellia japonica* Petals

Anthocyanins are key metabolites for coloration in plant organs, contributing to the purple leaves in *Brassica rapa* and *Brassica oleracea* (Zhang et al., 2014b; Horiuchi et al., 2020), and the purple skin in *Capsicum annuum* and *Solanum melongena* (Zhang et al., 2014c; Tang et al., 2020). Considering the health value of anthocyanins, foods rich in anthocyanins and anthocyanin extracts have broad market application prospects, and finding new sources of anthocyanin extraction has become a new problem. Similar to the flowers of other species, the petals of *C. japonic*a rich in anthocyanins. Thus, *C. japonica* may be served as a new source of natural anthocyanins extraction. The content of anthocyanins in the petals of *C. japonica* was determined in this study, and the results showed that the color intensity of the *C. japonica* petals was changed with the different anthocyanin contents (Figure 1). Here, in the red to dark-red petals of *C. japonica*, the anthocyanin content was greater than 20 mmol/g and even reached 85 mmol/g, which is significantly higher than the anthocyanin content of the peel extract of *S. melongena* (Todaro et al., 2009; Boulekbache-Makhlouf et al., 2013), indicating that *C. japonica* may have better application prospects as a natural food pigment.

There are many species of anthocyanins in colorful plants, of which six are well-known, namely cyanidin, delphinidin, peonidin, malvidin, pelargonidin, and petunidin (De Rosso and Mercadante, 2007). The molecular structures and chemical characteristics of these anthocyanins have been extensively investigated. Generally, cyanidin contributes to red-purple, delphinidin contributes to blue-red or purple, and pelargonidin contributes to orange and red (Khoo et al., 2017). In our study, only four aglycones of anthocyanin were detected in the *C. japonica* petals, namely cyanidin, pelargonidin, delphinidin, and peonidin. Malvidin and petunidin were not detected, indicating that these are not involved in the coloration of *C. japonica* petals. These findings corroborate the results of Xue et al. (2019), who demonstrated that cyanidin, pelargonidin, and delphinidin were the main components of the red flowers in strawberries. Interestingly, the results of this study differ from those of Zhou et al. (2020), who found that cyanidin, malvidin, petunidin, and pelargonidin were the main anthocyanin components in the pink flowers of *Camellia sinensis* (same genus species), which may be the results of domestication because *C. sinensis* is used as a drink rather than a decoration plant.

Anthocyanins isolated in nature are highly unstable and susceptible to degradation, resulting in the loss of biological activity and discoloration. Therefore, anthocyanins in organisms are bound to glycosides to enhance their stability (Ongkowijoyo et al., 2018). In this study, five types of glycoside were detected by analyzing the molecular structure of the anthocyanins, including two monoglycosides (glucose and arabinose) and three polyglycosides (di-glucoside, rutinose, and sambubioside). The data showed that anthocyanins containing polyglycosides accounted for 17.14–39.11% of the total anthocyanins in the *C. japonica* samples. Moreover, anthocyanins in the form of syringic acid (cyanidin-*O-*syringic acid) accounted for a maximum of 3.25% of total anthocyanins, which have also been reported to result in the rubellis mutant tepals of *Michelia maudiae* (Lang et al., 2019). However, in strawberries, although the content of anthocyanins varies among species, most anthocyanins in strawberries (more than 94%) are monoglycosides (Kelebek and Selli, 2011). The binding positions of the monoglycoside and polyglycoside anthocyanins are different. According to the report of Du et al. (2018), monoglycoside anthocyanins naturally occur at the 3- position, but when more than one glucose, they can also be associated with the 5-hydroxyl position. Anthocyanin degradation begins with the separation of a sugar molecule from the anthocyanin structure, and glycan substituents of anthocyanins are more readily lost from the 5- position than the 3- position (De Rosso and Mercadante, 2007). Therefore, polyglycoside anthocyanins exhibit stronger stability than monoglycoside anthocyanins. These findings suggest that polyglycoside anthocyanins are abundant and potentially of greater commercial value in *C. japonica*.

### Cyanidin-3-*O-*(6*^″^*-*O-*malonyl) Glucoside Content Contributes to Flower Color Variation in *Camellia japonica*

Differences in flower color are typically caused by different anthocyanin types and contents. High anthocyanin content in petals can make flowers darker in color. For example, anthocyanins are not present in white flower mutants of *Gentiana triflora*, but do accumulate at high levels in the blue flowers of *G. triflora* (mainly delphinidin) (Nakatsuka et al., 2005). In this study, 11 DAAs among CK vs T1, CK vs T2, CK vs T3, and CK vs T4 were identified according to the anthocyanin content in each sample, which indicated that these 11 anthocyanins contribute to the different petal colors in *C. japonica*. By analyzing the relative contents of these anthocyanins in the five groups, the results found that the content of cyanidin-3-*O-*(6^″^-*O-*malonyl) glucoside varied greatly among T1, T2, T3, and T4. However, cyanidin-3-*O-*(6^″^-*O-*malonyl) glucoside was almost undetectable in CK, which indicated that cyanidin-3-*O-*(6^″^-*O-*malonyl) glucoside is the main source of pink, red, and crimson phenotypes (Figure 2D). This finding supports the results of Cheng et al. (2014), who proposed that cyanidin-3-glucoside is the primary component in the red flowers of *Prunus persica*. Reports on *C. sinensis* document that cyanidin *O-*syringic acid, petunidin 3-*O-*glucoside, and pelargonidin 3-*O-*β-d-glucoside accumulate in pink flower but not in white flower, which indicates that these compounds play important role in the color expressing of the pink flower (Zhou et al., 2020). Moreover, cyanidin-3-*O-*rutinoside, peonidin-3-*O-*glucoside, cyanidin-3-*O-*glucoside, and pelargonidin-3-*O-*glucoside greatly accumulated in T1, T2, T3, and T4, implying that these anthocyanins play important roles in deepening the color of *C. japonica* petals (Figure 2D).

### Key Structural Genes Responsible for Anthocyanin Synthesis in *Camellia japonica* Petals

The anthocyanin biosynthesis pathway was reported as early as 1995 (Holton and Cornish, 1995), and the functions of key genes in the pathway have been explored in recent studies. For example, Zhang Y. et al. (2020) found that the six-base insertion mutation of *PsF3*′*H* upregulated the expression of *PsFLS*, resulting in difference in anthocyanin accumulation and acyanic flowers in *P. suffruticosa*. Considering the difference in anthocyanin contents among these *C. japonica* petals, two genes (*CjANS*, *CSS0029211*; *Cj4CL*, *CSS0016246*) among the 37 DEGs in the anthocyanin synthesis pathway were identified. We speculate that these two genes may play key roles in anthocyanin accumulation (Figure 4). As one of the key enzyme genes in the downstream of the anthocyanin biosynthesis pathway, *ANS* catalyzes the transformation of leucoanthocyanidin to a colored anthocyanidin before the final glycosylation steps (Hichri et al., 2011). Li et al. (2019) reported that overexpression of *SmANS* enhanced anthocyanin accumulation and resulted in the purple-red phenotype of *S. miltiorrhiza*, indicating the important function of *ANS* in anthocyanin biosynthesis. In *Camellia nitidissima* Chi., Zhou et al. (2017) found that *CnANS* regulated the accumulation of flavonols and anthocyanin, thus contributing to the golden flower of *C. nitidissima*. Moreover, the expression of *CnCHS*, *CnF3H*, and *CnFLS* also play critical roles in the formation of golden flowers (Zhou et al., 2017). Reports in *Arabidopsis thaliana* documented that the anthocyanin content reduced by 70% in the mutant of *At4CL3* compared to the wild type, which confirmed the function of *4CL* in anthocyanin accumulation (Li et al., 2015). Therefore, we speculate that the differential expression of *CjANS* and *Cj4CL* is an important contributor to the petal color diversity in *C. japonica* in this study.

### Transcription Factors Involved in Anthocyanin Synthesis in *Camellia japonica* Petals

Except to structural genes, TFs also play important roles in anthocyanin biosynthesis, such as *MYB*, *bHLH*, *WD protein*, and *MADS-box* (Zhang et al., 2014a). In *P. persica*, *PpNAC1* promotes anthocyanin accumulation by activating the transcription of *PpMYB10.1*, while the expression of *PpNAC1* is repressed by *PpSPL1* (Zhou et al., 2015). The upstream regulatory network of *SPL* was explored by Wang et al. (2020), who confirmed that the function of *SPL* in regulating anthocyanin biosynthesis was targeted by *miR156*, *miR858*, and *miR160h*. Thus, anthocyanin biosynthesis in plants involves a complex network that is not only the function of a single structural gene or TF but also the co-functioning of multiple TFs and structural genes (Jian et al., 2019). In this study, a large number of TFs were identified by analyzing the transcriptome annotation results. Based on the expression level of TFs obtained from the transcriptome data, 19 TFs (detailed in Table1 and Supplementary Figure 4) were found to highly associate with the total anthocyanin content (| PCC| ≥ 0.9), and these TFs may have an essential function in the phenotypic expression of petal color. Interestingly, among these TFs, three *CjMYBs* showed two different expression patterns. The expression level of *CjMYB* (*CSS0015300*) was highest in CK, followed by T1, T2, T3, and T4, which was contrary to the total anthocyanin content trend. Conversely, the expression of two *CjMYBs* (*CSS0014780* and *CSS0045979*) exhibited a similar trend to the total anthocyanin content in the *C. japonica* petals, indicating that *CjMYB* (*CSS0015300*) negatively regulated anthocyanin accumulation, whereas *CjMYBs* (*CSS0014780* and *CSS0045979*) may positively regulate anthocyanin accumulation in *C. japonica*. In plants, MYB often forms protein complexes with bHLH and WD to participate in anthocyanin biosynthesis rather than regulate anthocyanin biosynthesis directly (Feng et al., 2020). Subsequently, two negatively correlated *CjbHLHs* (*MSTRG.36794* and *CSS0025568*) and three positively correlated *CjbHLHs* (*CSS0019162*, *CSS0029144*, and *CSS0018697*) were identified. Whether these *CjbHLH* TFs interact with *CjMYB* to regulate anthocyanin biosynthesis in *C. japonica* remains to be further investigated.

### Other Key Candidate Genes Related to Anthocyanin Synthesis in *Camellia japonica* Petals

The formation of anthocyanins depends on glycosylation, hydroxylation, acylation, and methoxylation to maintain stability, and this process is controlled by some transporters and other proteins (Jiang et al., 2020). In this study, key genes involved in the coloring of *C. japonica* petals were identified by WGCNA, and 16 genes exhibiting the highest correlation were selected to construct a directed interaction network diagram. The results showed that these 11 anthocyanins were regulated by one to six genes and only cyanidin-3-*O-*(6^″^-*O-*p-Coumaroyl) glucoside was negatively regulated. The annotation results of the 16 genes confirmed the regulation of certain genes in anthocyanin synthesis has been reported in other species. Wei et al. (2019) confirmed that *CsGSTF11* was promoted by *CsMYB75* and resulted in anthocyanin hyperaccumulation in purple tea. In this study, two *CjGSTFs* (*CjGSTF11* and *CjGSTF12*) identified by WGCNA may have similar functions in the hyperaccumulation of anthocyanin in *C. japonica* petals. The glycosylation of anthocyanins was catalyzed by *UGTs* (the members of GT family), which is the final step in anthocyanin biosynthesis and leads to diverse anthocyanin molecules in *A. thaliana* (Li et al., 2017). The study of UGTs in *A. thaliana* showed that *UGT79B2* and *UGT79B3* elevate various stress tolerance by modulating anthocyanin accumulation (Li et al., 2017). In the WGCNA, three *UGTs* (*CjUGT73A*, *CjUGT87A*, and *CjUGT71A*) and one *CjGT* were identified to target two, two, one, and four types of anthocyanins, respectively, indicating that these *GTs* are responsible for anthocyanin glycosylation in *C. japonica* petals. Moreover, one zinc-finger protein (*CjGRP2*) and two sugar transporters (*CjERD6L1* and *CjERD6L2*) were identified, and their functional regulation in anthocyanin synthesis in other species has been reported (Jeong et al., 2010; Shi et al., 2018). Taken together, these results indicate that these 16 genes might play important roles in the color diversity in the *C. japonica* petals.

## Conclusion

In this study, metabolome and transcriptome analyses were used to identify key anthocyanins and genes responsible for *C. japonica* petal coloration. Overall, a total of 187 flavonoid metabolites were detected in *C. japonica* petals, including 25 anthocyanins. Cyanidin-3-*O-*(6^″^-*O-*malonyl) glucoside was the main anthocyanin component in the *C. japonica* petals, while cyanidin-3-*O-*rutinoside, peonidin-3-*O-*glucoside, cyanidin-3-*O-*glucoside, and pelargonidin-3-*O-*glucoside were responsible for the color intensity of the *C. japonica* petals. Moreover, 37 DEGs (especially *ANS* and *4CL*) in the anthocyanin biosynthesis pathway, 19 differentially expressed TFs (such as *GATA*, *MYB*, *bHLH*, *WRKY*, and *NAC*), and 16 other regulators (including two *GSTFs*, four *GTs*, two sugar transporters, and one zinc-finger protein) were identified as candidate regulators contributing to the color diversity of *C. japonica* petals. The results of this study provide valuable information and new insights for further evaluation on the genetic diversity of *C. japonica*.

## Data Availability Statement

The datasets presented in this study can be found in online repositories. The names of the repository/repositories and accession number(s) can be found below: Genome Sequence Archive in National Genomics Data Center, China National Center for Bioinformation/Beijing Institute of Genomics, Chinese Academy of Sciences; (Wang et al., 2017; CNCB-NGDC Members and Partners, 2021) CRA003840.

## Author Contributions

FX and MyF designed the research. MyF wrote the manuscript. MyF, XY, JrZ, and JbY collected the experimental materials. XY, LW, and YT accomplished the experiment involved in this manuscript. XyY helped for the data analyzing. SyC, WwZ, and YlL offered the key idea and scientific guidance for this research. FX revised the manuscript critically for important intellectual content. MyF sorted out and analyzed all the materials in this research. All authors listed here contributed and approved the manuscript.

## Conflict of Interest

The authors declare that the research was conducted in the absence of any commercial or financial relationships that could be construed as a potential conflict of interest.

## Footnotes

1 www.anpel.com.cn/cnw

2 http://www.anpel.com.cn/

3 www.shimadzu.com.cn/

4 www.appliedbiosystems.com.cn/

5 http://tpia.teaplant.org/

6 https://international.biocloud.net/zh/dashboard

7 http://www.omicshare.com/tools

8 https://en.tiangen.com/about/index.html

9 http://www.vazyme.com/Home.html

10 https://sg.idtdna.com/pages

## Supplementary Material

Supplementary Figure 1 Sample correlation analysis and comparative analysis of DEGs. **(A)** Sample relation analysis using FPKM value of all genes. Red represents a higher similarity. **(B)** Venn analysis among CK vs T1, CK vs T2, CK vs T3, and CK vs T4. **(C)** Number of DEGs among all comparison units.

Supplementary Figure 2 The GO and KEGG enrichment results of overlapping DEGs (634 DEGs). **(A)** GO enrichment results of overlapping DEGs. **(B)** The enrichment results of the top 15 KEGG pathways according to the p value. Red presents significant enrichment.

Supplementary Figure 3 The identification results of TFs related to anthocyanin accumulation. **(A)** The number and type of TFs and differentially expressed TFs. **(B)** The heatmap analysis of TFs involved in anthocyanin accumulation according to FPKM value. The red represents high expression level, while blue represents low expression level.

Supplementary Figure 4 The expression level of DEGs in different modules. **(A)** The expression trend of genes in brown module among all samples. **(B)** The expression trend of genes in turquoise module among all samples. **(C)** The expression trend of genes in yellow module among all samples.

Supplementary Figure 5 qRT-PCR validation of gene expression level in the transcriptome. The color of columns represents different samples. The height of column represents qRT-PCR results of genes (2^–Δ^ ^Δ^ ^*Ct*^), while the line represents FPKM value of genes.

Supplementary Figure 6 Correlation analysis of the results between RNA-seq and qRT-PCR. The qRT-PCR (2^–Δ^ ^Δ^ ^*Ct*^) and RNA-seq results were normalized by log_2_.

Supplementary Table 1 Primers used in qRT-PCR.

Supplementary Table 2 The classification and quantification results of all detected metabolites.

Supplementary Table 3 Summary of sequencing data.

Supplementary Table 4 Detection of sample alignment rate.

Supplementary Table 5 Evaluation of sample alignment area.

Supplementary Table 6 Assessment of sample alignment area.

## Abbreviations

PAL: phenylalanine ammonia-lyase
4CL: 4-coumaroyl CoA ligase
CYP73A: *trans-*cinnamate 4-monooxygenase
CHS: chalcone synthase
CHI: chalcone isomerase
F3H: flavonone 3-hydroxylase
F3 ′ H: flavonoid 3 ′ -monooxygenase
FLS: flavonol synthase
F3 ′ 5 ′ H: flavonoid 3 ′, 5 ′ -hydroxylase
DFR: dihydroflavonol 4-reductase
LAR: leucoanthocyanidin reductase
ANS: anthocyanidin synthase
UFGT: anthocyanidin 3-*O-*glucosyltransferase
GSTF: glutathione S-transferase
UGT: UDP-glycosyltransferase
ERD6L: sugar transporter ERD6-like
GRP2: glycine-rich RNA-binding protein 2
VTC: GDP-L-galactose phosphorylase/guanyltransferase
HSP: heat-shock protein
CST12: cytosolic sulfotransferase 12
lecRLK3: lectin S-receptor -like serine/threonine-protein kinase 3
BCCP: biotin carboxyl carrier protein of acetyl-CoA carboxylase.
