# Supplementary material for: Unraveling the Regulatory Mechanism of Color Diversity in Camellia japonica Petals by Integrative Transcriptome and Metabolome Analysis
Source: Front Plant Sci. 2021 Jun 11;12:685136. doi: 10.3389/fpls.2021.685136 (PMC8226227; doi:10.3389/fpls.2021.685136)
Supplement: Supplementary Figure 1 — Sample correlation analysis and comparative analysis of DEGs. (A) Sample relation analysis using FPKM value of all genes. Red represents a higher similarity. (B) Venn analysis among CK vs T1, CK vs T2, CK vs T3, and CK vs T4. (C) Number of DEGs among all comparison units. [file Data_Sheet_1.docx]

***Supplementary Material***

**Supplementary Table S1. Primers used in qRT-PCR.**

| **Gene ID** | **Primer** | **Primer sequence (5’- 3’)** |
| --- | --- | --- |
| *CSS0007059* | Sense | GCCGTGGAATCTCTGTTTCT |
|  | Anti-sense | GTTGTGTAATTGCCCTACCTTTG |
| *CSS0007873* | Sense | CAACAACCCATGTACCGTTTAC |
|  | Anti-sense | ATAAGCATCTGGGCACCTATC |
| *CSS0011810* | Sense | ACACCTGAGGAGGAGTACAA |
|  | Anti-sense | CTCCGGGTCTTTCTTAGACTTATG |
| *CSS0020564* | Sense | CCTGAGGGTACAGAGAGAACTA |
|  | Anti-sense | CGGGCTATATCCCAAGACAAA |
| *CSS0022874* | Sense | GGCGTGGATCATCAACTTACA |
|  | Anti-sense | AGGAGGCACCATCACAATAAC |
| *CSS0049675* | Sense | TTGAAGGGTACCGGCATAAC |
|  | Anti-sense | AGCATCAGCCAACCTCTTC |
| *CSS0018697* | Sense | CAGACAACCACAAGCTCTTCTA |
|  | Anti-sense | CATTGCGAAGGGTGTGTAAATC |
| *CSS0021462* | Sense | GGTTCCAGGACCAATCTTCTT |
|  | Anti-sense | CTACCTCCTTCTCTTTGCCTTC |
| *CSS0002094* | Sense | CATCCAAGGACCGTGAAGAATA |
|  | Anti-sense | CCAAGCAGATAAGCCGAGTAA |
| *CSS0005784* | Sense | CTGTGTCTGTTGCTGAGTATCC |
|  | Anti-sense | TCGTACAAGCGCATCAAAGT |
| *CSS0013223* | Sense | GCTGTCCTGGAACTACAACTAC |
|  | Anti-sense | CCTTGAACGATACGAGTGAATCT |
| *CSS0030581* | Sense | AAGGTGCAGATGTGGTTGAG |
|  | Anti-sense | GAGAACAGGGAGTGGCTTATTG |
| *CSS0037714* | Sense | CCTAGTGATTCTGGTGGTGATG |
|  | Anti-sense | GTGTCCAGTCTACCTTCATCTTC |
| *CSS0050363* | Sense | TATGTTGGGAAGTGGTGTGAAG |
|  | Anti-sense | CTGCTGGAGAGAGCCTTAGATA |
| *CSS0000220* | Sense | GGAAGGTGTTCCTTTAGGAGTG |
|  | Anti-sense | TGCCACTTTCCTTCTCCATTAG |
| *CSS0023103* | Sense | CTCTGTGAGGCTTTGTCTCTT |
|  | Anti-sense | CGCATATGTGGTGGTTGATTG |
| *CSS0026012* | Sense | CGCCTCCGTTGGATCAATTA |
|  | Anti-sense | GAGGAGGCTGTGGAGTTTAATG |
| *CSS0028896* | Sense | CACCTCCTGCTTTGGATGAA |
|  | Anti-sense | GGGAGACATAAGCGGGAAATAG |
| *CSS0040328* | Sense | GCTCAGAATTAGCCCTCCTTATC |
|  | Anti-sense | ATCTCCACAACTGCACTCTTC |
| *CSS0049966* | Sense | GGCTGCCATAGCTTCTTACTTA |
|  | Anti-sense | CATCAAAGGCTTCTTGCTCTTG |
| *CSS0017161* | Sense | GGAGACGCAGGAAAGAAAGAA |
|  | Anti-sense | TCCTCCACACCATCGAAATAAC |
| *CSS0023105* | Sense | GAAGCTATGGAGGAGTGTCATAAA |
|  | Anti-sense | CTGGAGGGATCTTTGATGTGAG |
| *CSS0024640* | Sense | GGAGGTGCCGTTTCTGATAAT |
|  | Anti-sense | AACCCTAATCGTCTTGTCCTTG |
| *CSS0029115* | Sense | CAAACTCTTACTCTCCGCCTAC |
|  | Anti-sense | AGGAGATTTGAGGTCGAGGA |
| *CSS0042150* | Sense | CGGATCATCTCTTACGGTGAAC |
|  | Anti-sense | CATCCTTCATCCAACCCTTCTC |

**Table S1-Continued.**

| CSS0004477 | Sense | CTTTGGAACGGTAGCGAAATTG |
| --- | --- | --- |
|  | Anti-sense | GTCTCCCAAGGACCAAAGAAA |
| *CSS0045518* | Sense | GAGACCATCACGGAAGTACATAG |
|  | Anti-sense | GAGAGATTGCCTGCTGAAGA |
| *CSS0003013* | Sense | GGCTATGTAGACGACGATGATG |
|  | Anti-sense | GATGGGTGGCTCACAAGAA |
| CSS0026138 | Sense | TGAGGAAATAGAGCCCGAAATC |
|  | Anti-sense | CCGTTCCAAAGCCAATGTATG |
| CSS0002506 | Sense | CAACTGGAAGAACCCAGAAGAG |
|  | Anti-sense | CACCAAACGGAAGGTACCTAAA |
| CSS0003562 | Sense | GGTGAAGCCAGAAGAGATCAA |
|  | Anti-sense | GTAAGCAGGCAATCTTGGTTTAG |
| CSS0004788 | Sense | CCACTTGAGCCATACCTTTCT |
|  | Anti-sense | CCCTATGAAGCTCCCTGTTATG |
| CSS0006278 | Sense | GTGAATGGGCTGAGGCTTTA |
|  | Anti-sense | TCACGCATGGTTTCAGGATAG |
| CSS0008432 | Sense | CATCAGCTAAGAGGTGGATGAA |
|  | Anti-sense | GTGACCATGAACAGCATCAATC |
| CSS0009572 | Sense | TCGTGTCCGCGATGATAAAG |
|  | Anti-sense | ACCACAGATACCAAGTCCAAAT |
| CSS0015732 | Sense | CTGCAACTCTTCGTCTCTTCT |
|  | Anti-sense | GATTATCCGGGTGATCCTTCTC |
| CSS0036213 | Sense | CCACCCAGATTGAGAACACTAC |
|  | Anti-sense | GAGGTGAATAGGAGGGTGTTTG |
| MSTRG.32318 | Sense | GCAACAGGGTCAAGTCCTATTA |
|  | Anti-sense | GCCATAGTAGAGGTGGAAAGAC |
| MSTRG.39374 | Sense | CATCATGGAGAACCCTGAAGATAG |
|  | Anti-sense | GGAGCGAGAAGTAGCTGTAATG |
| MSTRG.42910 | Sense | CCTAAGTCCACTAGCCGTAAAC |
|  | Anti-sense | CGAGATGGTTGTGACTCTTCTT |
| MSTRG.18301 | Sense | GTACTCCACCACTTCCTGTATG |
|  | Anti-sense | AGCGTCCCTGTGAATCAATATC |
| Cj18SrRNA | Sense | TCTCAACCATAAACGATGCCGACCAG |
|  | Anti-sense | TTTCAGCCTTGCGACCATACTCCC |

**Supplementary Table S2. The classification and quantification results of all detected metabolites.**

| **Compounds** | **Class I** | **Class II** | **Samples** | | | | | | | | | | | | | | |
| --- | --- | --- | --- | --- | --- | --- | --- | --- | --- | --- | --- | --- | --- | --- | --- | --- | --- |
|  |  |  | **CK1** | **CK2** | **CK3** | **T1-1** | **T1-2** | **T1-3** | **T2-1** | **T2-2** | **T2-3** | **T3-1** | **T3-2** | **T3-3** | **T4-1** | **T4-2** | **T4-3** |
| Pelargonidin-3-O-glucoside | Flavonoids | Anthocyanins | 9 | 9 | 9 | 1750000 | 1370000 | 1170000 | 7110000 | 7780000 | 9010000 | 2070000 | 2640000 | 2270000 | 27700000 | 28200000 | 26200000 |
| Delphinidin-3-O-arabinoside | Flavonoids | Anthocyanins | 6780 | 3450 | 3620 | 10100 | 6980 | 10000 | 11500 | 10100 | 8750 | 14000 | 19100 | 18900 | 22000 | 24200 | 19200 |
| Cyanidin-3-O-glucoside (Kuromanin)* | Flavonoids | Anthocyanins | 9 | 9 | 9 | 2460000 | 2030000 | 2140000 | 15900000 | 18200000 | 18000000 | 4040000 | 3950000 | 3890000 | 49800000 | 49900000 | 47300000 |
| Peonidin-3-O-glucoside | Flavonoids | Anthocyanins | 9 | 9 | 9 | 945000 | 861000 | 901000 | 20200000 | 23400000 | 22300000 | 8940000 | 8950000 | 9530000 | 61500000 | 69800000 | 56600000 |
| Cyanidin-O-syringic acid | Flavonoids | Anthocyanins | 18100 | 6790 | 10000 | 399000 | 291000 | 306000 | 3060000 | 3860000 | 3770000 | 788000 | 572000 | 547000 | 9190000 | 9320000 | 9380000 |
| Delphinidin-3-O-(6''-O-acetyl) glucoside | Flavonoids | Anthocyanins | 81600 | 107000 | 88700 | 13100 | 11800 | 13700 | 3530 | 3250 | 4130 | 9 | 9 | 9 | 40800 | 18800 | 33800 |
| Cyanidin-3-O-(6''-O-malonyl) glucoside | Flavonoids | Anthocyanins | 28700 | 17100 | 15700 | 841000 | 806000 | 795000 | 4220000 | 4550000 | 4290000 | 3910000 | 4260000 | 4670000 | 1560000 | 1860000 | 1560000 |
| Malvidin-3-O-(6''-O-malonyl) glucoside | Flavonoids | Anthocyanins | 110000 | 149000 | 130000 | 140000 | 134000 | 140000 | 176000 | 153000 | 177000 | 107000 | 142000 | 138000 | 114000 | 135000 | 121000 |
| Pelargonidin-3-O-rutinoside | Flavonoids | Anthocyanins | 4950000 | 5850000 | 5170000 | 7300000 | 6820000 | 7430000 | 4450000 | 4040000 | 3920000 | 4390000 | 5500000 | 5840000 | 1530000 | 1750000 | 1640000 |
| Cyanidin-3-O-sambubioside [Cyanidin-3-O-(2''-O-xylosyl) glucoside] | Flavonoids | Anthocyanins | 9 | 9 | 9 | 2350000 | 2060000 | 2400000 | 9650000 | 8500000 | 9030000 | 25500000 | 18100000 | 21500000 | 620000 | 609000 | 620000 |
| Cyanidin-3-O-(6''-O-p-Coumaroyl) glucoside | Flavonoids | Anthocyanins | 132000 | 63600 | 41400 | 5700000 | 5590000 | 5440000 | 8390000 | 9450000 | 8030000 | 10600000 | 11600000 | 12500000 | 8950000 | 8450000 | 9270000 |
| Pelargonidin-3,5-O-diglucoside | Flavonoids | Anthocyanins | 125000 | 144000 | 256000 | 70500 | 88800 | 77800 | 35900 | 41200 | 31400 | 36000 | 46000 | 44500 | 24800 | 15300 | 30100 |
| Cyanidin-3-O-rutinoside (Keracyanin) | Flavonoids | Anthocyanins | 9 | 9 | 9 | 1690000 | 1400000 | 1340000 | 12500000 | 15100000 | 15000000 | 15600000 | 15900000 | 15500000 | 61900000 | 64700000 | 67000000 |
| Peonidin-3-O-(6''-O-p-Coumaroyl) glucoside | Flavonoids | Anthocyanins | 355000 | 279000 | 272000 | 3530000 | 2890000 | 3200000 | 5780000 | 6100000 | 6040000 | 13600000 | 12100000 | 16500000 | 9470000 | 9610000 | 9830000 |
| Cyanidin-3-O-(6''-O-caffeoyl) glucoside* | Flavonoids | Anthocyanins | 444000 | 165000 | 173000 | 23200000 | 20400000 | 22100000 | 54600000 | 71100000 | 60300000 | 125000000 | 140000000 | 139000000 | 21600000 | 14800000 | 18400000 |
| Delphinidin-3-O-(6''-O-p-coumaroyl) glucoside* | Flavonoids | Anthocyanins | 4190000 | 4760000 | 7760000 | 3930000 | 3930000 | 4020000 | 2120000 | 1960000 | 1800000 | 7330000 | 8390000 | 8700000 | 4050000 | 4490000 | 4550000 |
| Delphinidin-3-O-(6''-O-caffeoyl) glucoside | Flavonoids | Anthocyanins | 35900 | 32700 | 41800 | 10400 | 14100 | 8440 | 115000 | 133000 | 95400 | 78500 | 33000 | 55400 | 9 | 9 | 9 |
| Cyanidin-3-O-(6''-O-p-coumaroyl-2''-O-xylosyl) glucoside | Flavonoids | Anthocyanins | 2790000 | 2870000 | 3270000 | 31300000 | 36400000 | 36100000 | 4670000 | 4590000 | 4920000 | 20500000 | 17500000 | 18500000 | 29900000 | 25700000 | 21500000 |
| Cyanidin-3-O-(6''-O-caffeoyl-2''-O-xylosyl) glucoside | Flavonoids | Anthocyanins | 9 | 9 | 9 | 65900 | 45900 | 78000 | 395000 | 342000 | 265000 | 1420000 | 1060000 | 1690000 | 9 | 9 | 9 |
| Cyanidin-3-O-(6-O-(E)-p-coumaroyl-glucoside)-5-O-glucoside | Flavonoids | Anthocyanins | 454000 | 321000 | 382000 | 12900000 | 13000000 | 12900000 | 1200000 | 912000 | 1150000 | 2980000 | 2610000 | 2240000 | 1610000 | 1840000 | 1350000 |
| Delphinidin-3-O-(2'''-O-p-coumaroyl) rutinoside | Flavonoids | Anthocyanins | 131000 | 112000 | 172000 | 172000 | 170000 | 140000 | 80000 | 82000 | 79200 | 245000 | 294000 | 359000 | 302000 | 326000 | 307000 |
| Delphinidin-3-O-rutinoside-7-O-glucoside | Flavonoids | Anthocyanins | 12300 | 17700 | 7480 | 12200 | 13700 | 9460 | 8220 | 15800 | 11800 | 4810 | 5880 | 7210 | 4740 | 6090 | 7250 |
| Cyanidin-3-O-[6''-O-(Z)-p-coumaroyl-2''-O-xylosyl] glucoside-5-O-glucoside | Flavonoids | Anthocyanins | 9 | 9 | 9 | 22600 | 25000 | 24400 | 7830 | 14200 | 9640 | 8590 | 5770 | 12700 | 57200 | 60100 | 54200 |
| Cyanidin-3-O-[6''-O-(E)-p-coumaroyl-2''-O-xylosyl] glucoside-5-O-glucoside | Flavonoids | Anthocyanins | 73300 | 36600 | 35400 | 630000 | 599000 | 485000 | 181000 | 132000 | 101000 | 47300 | 28600 | 20100 | 9 | 9 | 9 |
| Cyanidin-3-O-rutinoside-5,3'-di-O-glucoside | Flavonoids | Anthocyanins | 8020 | 6870 | 7890 | 65300 | 54500 | 85400 | 18900 | 21700 | 20900 | 7130 | 8910 | 7500 | 12900 | 14400 | 12300 |
| 3,3',4',5,7-Pentahydroxyflavan(4→8)-3,3',4',5,7-pentahydroxyflavan;(2R,2'R,3R,3'R,4R)-form,3'-O-(3,4,5-Trihydroxybenzoyl) | Flavonoids | Biflavones | 373000 | 321000 | 427000 | 280000 | 240000 | 310000 | 52700 | 83600 | 94100 | 41600 | 80100 | 75500 | 143000 | 286000 | 190000 |
| Naringenin chalcone* | Flavonoids | Chalcones | 21900 | 23300 | 19900 | 26600 | 34100 | 31600 | 64700 | 66900 | 56000 | 29800 | 32300 | 58900 | 180000 | 169000 | 203000 |
| Phloretin-2'-O-glucoside (Phlorizin) | Flavonoids | Chalcones | 825000 | 926000 | 799000 | 105000 | 108000 | 124000 | 976000 | 1040000 | 889000 | 527000 | 500000 | 495000 | 2070000 | 1980000 | 1730000 |
| Sieboldin | Flavonoids | Chalcones | 58700 | 61200 | 40900 | 20600 | 12900 | 18400 | 11800 | 3330 | 3770 | 5410 | 9230 | 36300 | 39000 | 31800 | 37000 |
| Naringenin (5,7,4'-Trihydroxy flavanone)* | Flavonoids | Dihydroflavone | 12100 | 13600 | 12800 | 16700 | 20400 | 17400 | 46600 | 47100 | 35800 | 15800 | 17800 | 32100 | 119000 | 116000 | 137000 |
| Eriodictyol (5,7,3',4'-Tetrahydroxyflavanone) | Flavonoids | Dihydroflavone | 36600 | 39000 | 33000 | 21200 | 18100 | 21200 | 31200 | 30400 | 33300 | 27600 | 51700 | 97000 | 173000 | 172000 | 185000 |
| Naringenin-7-O-glucoside (Prunin) | Flavonoids | Dihydroflavone | 4600000 | 4170000 | 3910000 | 3770000 | 3250000 | 5230000 | 3310000 | 3070000 | 3360000 | 2140000 | 3130000 | 3620000 | 2650000 | 3030000 | 2630000 |
| Eriodictyol-3'-O-glucoside* | Flavonoids | Dihydroflavone | 437000 | 440000 | 331000 | 119000 | 131000 | 124000 | 380000 | 341000 | 469000 | 123000 | 157000 | 337000 | 1160000 | 1190000 | 1070000 |
| Naringenin-7-O-Rutinoside (Narirutin)* | Flavonoids | Dihydroflavone | 408000 | 393000 | 373000 | 145000 | 127000 | 145000 | 357000 | 276000 | 303000 | 613000 | 669000 | 582000 | 320000 | 290000 | 322000 |
| Naringenin-7-O-Neohesperidoside (Naringin)* | Flavonoids | Dihydroflavone | 1370000 | 1400000 | 1380000 | 460000 | 481000 | 463000 | 1280000 | 974000 | 1040000 | 1960000 | 2360000 | 2170000 | 1130000 | 1020000 | 1150000 |
| Hesperetin-7-O-rutinoside (Hesperidin)* | Flavonoids | Dihydroflavone | 806000 | 750000 | 623000 | 740000 | 2460000 | 1140000 | 1410000 | 247000 | 256000 | 215000 | 477000 | 3860000 | 1590000 | 120000 | 1380000 |
| Hesperetin-7-O-neohesperidoside (Neohesperidin)* | Flavonoids | Dihydroflavone | 806000 | 687000 | 613000 | 713000 | 2770000 | 1300000 | 1430000 | 280000 | 288000 | 229000 | 492000 | 3980000 | 1570000 | 108000 | 1550000 |
| Pinobanksin* | Flavonoids | Dihydroflavonol | 13300 | 12500 | 12700 | 17200 | 19200 | 18200 | 44100 | 47300 | 35800 | 15500 | 17800 | 30700 | 120000 | 114000 | 140000 |
| Dihydrokaempferol | Flavonoids | Dihydroflavonol | 12700 | 10200 | 7360 | 18400 | 25600 | 26300 | 13600 | 12000 | 18600 | 11300 | 23800 | 17700 | 43900 | 47800 | 52300 |
| Dihydroquercetin (Taxifolin) | Flavonoids | Dihydroflavonol | 509000 | 635000 | 702000 | 477000 | 668000 | 530000 | 361000 | 399000 | 432000 | 172000 | 278000 | 440000 | 821000 | 756000 | 941000 |
| 3-O-Acetylpinobanksin | Flavonoids | Dihydroflavonol | 41500 | 48700 | 67400 | 142000 | 192000 | 263000 | 134000 | 114000 | 67700 | 168000 | 130000 | 237000 | 76800 | 65700 | 41000 |
| Dihydromyricetin (Ampelopsin) | Flavonoids | Dihydroflavonol | 22000 | 18700 | 18000 | 16800 | 14600 | 11700 | 9 | 9 | 9 | 20200 | 15000 | 24000 | 15100 | 13100 | 10500 |
| Hesperetin-5-O-glucoside | Flavonoids | Dihydroflavonol | 14300000 | 16400000 | 26500000 | 11100000 | 10800000 | 10600000 | 18300000 | 19000000 | 17700000 | 7460000 | 6370000 | 9040000 | 13100000 | 11500000 | 11500000 |
| Dihydromyricetin-3-O-glucoside | Flavonoids | Dihydroflavonol | 9 | 9 | 9 | 2000 | 2430 | 9 | 19200 | 29100 | 18200 | 20300 | 14300 | 28400 | 16200 | 13300 | 19300 |
| Phellamurin | Flavonoids | Dihydroflavonol | 441000 | 386000 | 175000 | 5230 | 7320 | 6140 | 62100 | 55100 | 87400 | 96700 | 64900 | 85200 | 53500 | 41300 | 39800 |
| Epiafzelechin | Flavonoids | Flavanols | 217000 | 180000 | 164000 | 399000 | 489000 | 482000 | 119000 | 58100 | 86800 | 20900 | 40500 | 34200 | 158000 | 177000 | 157000 |
| Catechin | Flavonoids | Flavanols | 1400000 | 1830000 | 1790000 | 1690000 | 2000000 | 2040000 | 1260000 | 1090000 | 1300000 | 1230000 | 1380000 | 1760000 | 1250000 | 1400000 | 1380000 |
| Epicatechin | Flavonoids | Flavanols | 8020000 | 8180000 | 8030000 | 9240000 | 8910000 | 9740000 | 8400000 | 8450000 | 7920000 | 6370000 | 6140000 | 6190000 | 7410000 | 8630000 | 7790000 |
| Epigallocatechin* | Flavonoids | Flavanols | 9 | 11700 | 5640 | 17000 | 20900 | 19500 | 143000 | 169000 | 140000 | 188000 | 276000 | 233000 | 118000 | 143000 | 154000 |
| Gallocatechin* | Flavonoids | Flavanols | 5420 | 6530 | 7520 | 25400 | 28300 | 19200 | 416000 | 510000 | 402000 | 293000 | 220000 | 201000 | 101000 | 88900 | 88300 |
| Catechin gallate* | Flavonoids | Flavanols | 3610000 | 3930000 | 1210000 | 1260000 | 1270000 | 1520000 | 364000 | 399000 | 379000 | 711000 | 2090000 | 230000 | 1250000 | 1400000 | 1070000 |
| Epicatechin gallate* | Flavonoids | Flavanols | 3640000 | 3550000 | 1260000 | 1150000 | 1280000 | 1580000 | 330000 | 335000 | 380000 | 665000 | 1960000 | 211000 | 1160000 | 1260000 | 1000000 |
| Catechin-(7,8-bc)-4β-(3,4-dihydroxyphenyl)-dihydro-2-(3H)-one | Flavonoids | Flavanols | 53400 | 170000 | 68600 | 16600 | 17600 | 22500 | 139000 | 206000 | 138000 | 364000 | 274000 | 345000 | 227000 | 270000 | 257000 |
| Catechin-(7,8-bc)-4α-(3,4-dihydroxyphenyl)-dihydro-2-(3H)-one | Flavonoids | Flavanols | 19300 | 99700 | 19800 | 8180 | 11900 | 10400 | 39300 | 60800 | 31500 | 174000 | 157000 | 239000 | 49100 | 64400 | 48000 |
| Epicatechin glucoside | Flavonoids | Flavanols | 1490000 | 1580000 | 2170000 | 2140000 | 2310000 | 2570000 | 1460000 | 1510000 | 1190000 | 2050000 | 2250000 | 2150000 | 1300000 | 1430000 | 1420000 |
| Epicatechin-epiafzelechin | Flavonoids | Flavanols | 1350000 | 1340000 | 1490000 | 3300000 | 3310000 | 3860000 | 1080000 | 1260000 | 1040000 | 976000 | 1040000 | 1140000 | 1810000 | 1810000 | 1850000 |
| Apiferol | Flavonoids | Flavonoid | 184000 | 206000 | 236000 | 83300 | 64100 | 88500 | 44600 | 36600 | 29300 | 70900 | 99700 | 338000 | 586000 | 528000 | 608000 |
| Isoscutellarein* | Flavonoids | Flavonoid | 9 | 9 | 9 | 2740 | 3730 | 2600 | 9 | 9 | 9 | 3040 | 3700 | 5550 | 15000 | 18000 | 17000 |
| Luteolin (5,7,3',4'-Tetrahydroxyflavone)* | Flavonoids | Flavonoid | 3580 | 2930 | 2360 | 5080 | 6890 | 4450 | 3280 | 2440 | 3740 | 4960 | 7280 | 11600 | 83000 | 95000 | 84300 |
| 3',4,4',5,7-Pentahydroxyflavan (Luteoforol) | Flavonoids | Flavonoid | 1110000 | 1420000 | 1140000 | 131000 | 345000 | 509000 | 276000 | 225000 | 146000 | 33700 | 82500 | 110000 | 283000 | 114000 | 229000 |
| Hispidulin (5,7,4'-Trihydroxy-6-methoxyflavone)* | Flavonoids | Flavonoid | 1290 | 1510 | 2060 | 13500 | 23600 | 13000 | 3730 | 2990 | 2240 | 10000 | 11900 | 23800 | 55300 | 58400 | 61700 |
| Diosmetin (5,7,3'-Trihydroxy-4'-methoxyflavone)* | Flavonoids | Flavonoid | 9 | 9 | 9 | 4890 | 6790 | 4800 | 2970 | 1960 | 1400 | 2670 | 4360 | 7840 | 15000 | 16500 | 19000 |
| 6,7,8-Tetrahydroxy-5-methoxyflavone | Flavonoids | Flavonoid | 1410 | 2240 | 2880 | 3670 | 11800 | 16900 | 13500 | 12700 | 3230 | 4910 | 7020 | 9120 | 3290 | 1690 | 2110 |
| Tricetin (5,7,3',4',5'-Pentahydroxyflavone) | Flavonoids | Flavonoid | 13200 | 10200 | 15500 | 8340 | 6940 | 8270 | 9030 | 7930 | 8340 | 3910 | 6430 | 11300 | 13100 | 8900 | 9240 |
| 4',5,7-Trihydroxy-3',6-dimethoxyflavone (Jaceosidin) | Flavonoids | Flavonoid | 2740 | 5810 | 6780 | 10900 | 37800 | 66500 | 21200 | 18500 | 5970 | 10500 | 16300 | 18900 | 9190 | 4930 | 5700 |
| 5,6,7,4'-Tetramethoxyflavone | Flavonoids | Flavonoid | 14100 | 11200 | 13800 | 16300 | 20600 | 37300 | 36900 | 4540 | 12000 | 3110 | 37200 | 63500 | 2730 | 2740 | 31900 |
| 5-Hydroxy-6,7,3',4'-tetramethoxyflavone | Flavonoids | Flavonoid | 20600 | 16100 | 15800 | 103000 | 91400 | 126000 | 29500 | 36100 | 10300 | 13900 | 26400 | 15400 | 8470 | 10100 | 4570 |
| 5-Hydroxy-6,7,8,3',4'-pentamethoxyflavone* | Flavonoids | Flavonoid | 5590 | 4610 | 7490 | 25500 | 17900 | 32600 | 35300 | 5090 | 3040 | 2540 | 33600 | 33600 | 9 | 3250 | 34200 |
| Nobiletin (5,6,7,8,3',4'-Hexamethoxyflavone) | Flavonoids | Flavonoid | 175000 | 157000 | 167000 | 148000 | 189000 | 350000 | 337000 | 51800 | 120000 | 43600 | 392000 | 618000 | 30200 | 31400 | 279000 |
| Kaempferol-3-O-arabinoside* | Flavonoids | Flavonoid | 72100 | 82900 | 91400 | 409000 | 379000 | 366000 | 116000 | 154000 | 108000 | 193000 | 229000 | 122000 | 9 | 9 | 9 |
| Galangin-7-O-glucoside* | Flavonoids | Flavonoid | 583000 | 562000 | 504000 | 154000 | 194000 | 150000 | 572000 | 507000 | 842000 | 252000 | 403000 | 92300 | 11400000 | 10700000 | 8810000 |
| Naringenin-4'-O-glucoside* | Flavonoids | Flavonoid | 600000 | 610000 | 525000 | 785000 | 905000 | 1200000 | 1100000 | 1180000 | 1290000 | 571000 | 691000 | 962000 | 1280000 | 1200000 | 1300000 |
| Luteolin-4'-O-glucoside* | Flavonoids | Flavonoid | 2360000 | 2460000 | 3770000 | 10600000 | 10300000 | 10500000 | 8220000 | 7220000 | 9690000 | 4550000 | 4690000 | 4200000 | 9 | 9 | 9 |
| Diosmetin-7-O-galactoside* | Flavonoids | Flavonoid | 450000 | 536000 | 381000 | 2080000 | 2520000 | 2240000 | 435000 | 472000 | 471000 | 902000 | 1180000 | 1090000 | 2850000 | 2860000 | 2730000 |
| Hesperetin-7-O-glucoside | Flavonoids | Flavonoid | 340000 | 475000 | 530000 | 665000 | 899000 | 826000 | 1380000 | 1280000 | 1220000 | 628000 | 744000 | 1240000 | 488000 | 9 | 9 |
| Taxifolin-3'-O-glucoside | Flavonoids | Flavonoid | 360000 | 424000 | 661000 | 249000 | 262000 | 242000 | 372000 | 432000 | 378000 | 167000 | 159000 | 174000 | 292000 | 193000 | 173000 |
| 3',5',5,7-Tetrahydroxy-4'-methoxyflavanone-3'-O-glucoside | Flavonoids | Flavonoid | 9 | 9 | 9 | 9 | 9 | 9 | 51500 | 54700 | 65200 | 23800 | 12600 | 26600 | 262000 | 280000 | 287000 |
| Tricin-7-O-Glucoside | Flavonoids | Flavonoid | 9 | 9 | 9 | 108000 | 125000 | 87600 | 9 | 9 | 9 | 2380 | 4370 | 2270 | 5750 | 11200 | 5560 |
| Tricin-7-O-saccharic acid | Flavonoids | Flavonoid | 3070000 | 2750000 | 3000000 | 863000 | 897000 | 944000 | 2310000 | 2450000 | 2620000 | 1370000 | 1700000 | 1330000 | 1680000 | 2120000 | 1750000 |
| Apigenin-7-O-(6''-p-Coumaryl) glucoside | Flavonoids | Flavonoid | 2260000 | 2220000 | 1910000 | 4640000 | 3930000 | 3540000 | 3450000 | 3770000 | 3470000 | 6990000 | 8040000 | 6450000 | 7320000 | 6890000 | 6530000 |
| Kaempferol-3-O-sambubioside | Flavonoids | Flavonoid | 189000 | 259000 | 278000 | 107000 | 113000 | 103000 | 87400 | 46800 | 78900 | 27300 | 19400 | 47900 | 40100 | 48200 | 22100 |
| Luteolin-7-O-(6''-caffeoyl) rhamnoside* | Flavonoids | Flavonoid | 9 | 9 | 9 | 1540000 | 1620000 | 1320000 | 1730000 | 2210000 | 1510000 | 3770000 | 3880000 | 4030000 | 2590000 | 2450000 | 2580000 |
| Luteolin-7-O-rutinoside | Flavonoids | Flavonoid | 9 | 9 | 9 | 336000 | 539000 | 630000 | 9 | 9 | 9 | 143000 | 146000 | 153000 | 217000 | 222000 | 174000 |
| Apigenin-6,8-di-C-glucoside* | Flavonoids | Flavonoid | 4860000 | 4710000 | 3450000 | 5830000 | 5660000 | 4790000 | 1130000 | 973000 | 1420000 | 1550000 | 2940000 | 973000 | 824000 | 1070000 | 991000 |
| Diosmetin-7-O-rutinoside (Diosmin) | Flavonoids | Flavonoid | 456000 | 295000 | 270000 | 4270000 | 3930000 | 4220000 | 8860000 | 9470000 | 7110000 | 16800000 | 16200000 | 22400000 | 11700000 | 12100000 | 12100000 |
| Kaempferol-3,7-di-O-glucoside* | Flavonoids | Flavonoid | 31800 | 14700 | 10800 | 2620000 | 2490000 | 3300000 | 2470000 | 2440000 | 3350000 | 20500000 | 17900000 | 18400000 | 2020000 | 1300000 | 2030000 |
| Luteolin-7-O-gentiobioside* | Flavonoids | Flavonoid | 30000 | 12000 | 19700 | 1890000 | 2360000 | 2670000 | 2480000 | 2790000 | 2540000 | 20400000 | 18300000 | 19900000 | 1650000 | 1520000 | 1700000 |
| Kaempferol-3-O-(2-O-Xylosyl-6-O-Rhamnosyl) Glucoside | Flavonoids | Flavonoid | 3030000 | 2790000 | 3340000 | 37400000 | 37600000 | 37600000 | 5230000 | 3740000 | 5980000 | 24600000 | 20800000 | 20300000 | 27200000 | 27900000 | 22600000 |
| Astragalin;2'',6''-Bis-O-(4-hydroxy-E-cinnamoyl) | Flavonoids | Flavonoid | 11900 | 6270 | 4270 | 82500 | 39800 | 69400 | 4230 | 2660 | 1630 | 28600 | 44600 | 19000 | 3770 | 20300 | 6640 |
| Kaempferol-3-O-(6''-Rhamnosyl-2''-Glucosyl) Glucoside (Camelliaside A)* | Flavonoids | Flavonoid | 548000 | 488000 | 547000 | 12100000 | 14400000 | 14200000 | 1310000 | 1320000 | 1290000 | 3080000 | 3240000 | 2760000 | 1790000 | 2220000 | 1680000 |
| Isohemiphloin | Flavonoids | Flavonoid carbonoside | 51500 | 96500 | 129000 | 19500 | 24400 | 21200 | 40800 | 20100 | 18900 | 38400 | 42400 | 58200 | 188000 | 198000 | 187000 |
| Luteolin-6-C-glucoside (Isoorientin) | Flavonoids | Flavonoid carbonoside | 54700 | 28200 | 58000 | 2200 | 10200 | 4550 | 9060 | 5720 | 3660 | 4540 | 14100 | 9 | 29000 | 23100 | 23000 |
| Schaftoside* | Flavonoids | Flavonoid carbonoside | 30500 | 29400 | 23600 | 22800 | 19300 | 12600 | 4050 | 3140 | 7410 | 16600 | 18500 | 7870 | 9 | 9 | 9 |
| Isoschaftoside* | Flavonoids | Flavonoid carbonoside | 2960000 | 3540000 | 2580000 | 1640000 | 1740000 | 1860000 | 794000 | 466000 | 833000 | 1140000 | 1920000 | 665000 | 9 | 9 | 9 |
| Apigenin-6-C-(2''-glucosyl) arabinoside* | Flavonoids | Flavonoid carbonoside | 6000000 | 5740000 | 5040000 | 3240000 | 3360000 | 2510000 | 1060000 | 892000 | 1660000 | 2630000 | 3090000 | 1150000 | 29800 | 31100 | 34200 |
| Apigenin-6-C-(2''-glucuronyl) xyloside | Flavonoids | Flavonoid carbonoside | 1380000 | 1790000 | 1680000 | 589000 | 789000 | 821000 | 541000 | 409000 | 552000 | 192000 | 226000 | 196000 | 422000 | 433000 | 395000 |
| Vitexin-2''-O-glucoside* | Flavonoids | Flavonoid carbonoside | 944000 | 785000 | 842000 | 817000 | 1050000 | 1000000 | 252000 | 227000 | 250000 | 327000 | 615000 | 175000 | 240000 | 163000 | 192000 |
| Phloretin-3,5-di-C-glucoside | Flavonoids | Flavonoid carbonoside | 59400 | 62500 | 69700 | 573000 | 532000 | 533000 | 950000 | 1220000 | 1020000 | 1140000 | 1090000 | 1140000 | 748000 | 667000 | 763000 |
| Quercetin* | Flavonoids | Flavonols | 9 | 9 | 9 | 93000 | 70600 | 117000 | 175000 | 257000 | 206000 | 157000 | 105000 | 196000 | 137000 | 128000 | 159000 |
| Morin* | Flavonoids | Flavonols | 189000 | 195000 | 283000 | 198000 | 228000 | 183000 | 252000 | 308000 | 738000 | 153000 | 181000 | 212000 | 398000 | 362000 | 421000 |
| Azaleatin (5-O-Methylquercetin) | Flavonoids | Flavonols | 12900 | 6060 | 9900 | 8100 | 9010 | 10800 | 5550 | 5730 | 7710 | 10800 | 23300 | 29700 | 2870 | 4600 | 6690 |
| Myricetin | Flavonoids | Flavonols | 28200 | 26000 | 14700 | 19500 | 19500 | 16100 | 14400 | 9730 | 7830 | 13700 | 15200 | 10800 | 31700 | 33800 | 31100 |
| Tangeretin | Flavonoids | Flavonols | 348000 | 358000 | 442000 | 508000 | 418000 | 1050000 | 1110000 | 107000 | 226000 | 88100 | 823000 | 1510000 | 90400 | 84900 | 777000 |
| 5-Hydroxyauranetin* | Flavonoids | Flavonols | 2090 | 1840 | 2970 | 10600 | 7650 | 13600 | 14700 | 2250 | 1310 | 869 | 13900 | 13300 | 390 | 1170 | 14400 |
| Kaempferol-3-O-arabinoside (Juglanin)* | Flavonoids | Flavonols | 19600 | 23400 | 26000 | 88400 | 93200 | 92600 | 36900 | 35600 | 28300 | 46900 | 73600 | 35700 | 9450 | 6160 | 3540 |
| Kaempferol-3-O-rhamnoside (Afzelin)(Kaempferin)* | Flavonoids | Flavonols | 20300 | 16100 | 6260 | 100000 | 94200 | 86000 | 9 | 9420 | 9 | 39300 | 30400 | 40100 | 9 | 9 | 9 |
| Quercetin-3-O-arabinoside (Guaijaverin)* | Flavonoids | Flavonols | 11700000 | 13600000 | 18600000 | 9490000 | 9160000 | 7800000 | 10200000 | 10400000 | 10400000 | 6900000 | 6390000 | 9880000 | 578000 | 632000 | 567000 |
| Morin-3-O-xyloside* | Flavonoids | Flavonols | 20600000 | 26000000 | 34700000 | 19100000 | 19600000 | 16700000 | 19700000 | 22200000 | 18900000 | 15000000 | 14300000 | 22800000 | 1300000 | 1360000 | 1240000 |
| Avicularin* | Flavonoids | Flavonols | 17800000 | 21600000 | 27700000 | 16100000 | 15800000 | 15200000 | 16400000 | 17700000 | 15600000 | 12200000 | 12500000 | 19900000 | 1020000 | 1110000 | 1030000 |
| Isorhamnetin-3-O-arabinoside* | Flavonoids | Flavonols | 70700 | 84600 | 128000 | 56900 | 57900 | 74200 | 42400 | 51200 | 41100 | 88500 | 107000 | 264000 | 9390 | 8540 | 6930 |
| Quercetin-3-O-rhamnoside (Quercitrin)* | Flavonoids | Flavonols | 47500 | 44400 | 70200 | 1650000 | 1740000 | 1360000 | 92700 | 88000 | 76800 | 1740000 | 1710000 | 2010000 | 29400 | 16700 | 29800 |
| Kaempferol-3-O-galactoside (Trifolin) | Flavonoids | Flavonols | 176000 | 235000 | 226000 | 592000 | 578000 | 658000 | 556000 | 460000 | 578000 | 254000 | 273000 | 207000 | 966000 | 750000 | 1310000 |
| Dihydrokaempferol-7-O-glucoside* | Flavonoids | Flavonols | 803000 | 988000 | 697000 | 232000 | 213000 | 224000 | 297000 | 277000 | 282000 | 395000 | 540000 | 677000 | 989000 | 904000 | 912000 |
| 8-Methoxykaempferol-7-O-rhamnoside* | Flavonoids | Flavonols | 3590 | 5380 | 2850 | 21600 | 17600 | 19700 | 9 | 9 | 9 | 18600 | 11600 | 30500 | 48400 | 39300 | 47700 |
| Quercetin-3-O-glucoside (Isoquercitrin)* | Flavonoids | Flavonols | 20000000 | 23500000 | 38800000 | 14800000 | 18200000 | 18100000 | 26300000 | 26700000 | 25300000 | 9950000 | 8850000 | 15700000 | 16300000 | 15900000 | 16700000 |
| 6-Hydroxykaempferol-7-O-glucoside* | Flavonoids | Flavonols | 3870000 | 3420000 | 4060000 | 4030000 | 3470000 | 3400000 | 2420000 | 2910000 | 2260000 | 6870000 | 4560000 | 7370000 | 5120000 | 7090000 | 5570000 |
| Quercetin-3-O-galactoside (Hyperin)* | Flavonoids | Flavonols | 25400000 | 29800000 | 43900000 | 19400000 | 23200000 | 21900000 | 31400000 | 31800000 | 34200000 | 13700000 | 11400000 | 18600000 | 21000000 | 20500000 | 20900000 |
| Isorhamnetin-3-O-Glucoside* | Flavonoids | Flavonols | 794000 | 746000 | 949000 | 939000 | 960000 | 898000 | 532000 | 587000 | 554000 | 811000 | 1220000 | 1940000 | 609000 | 639000 | 561000 |
| Rhamnetin-3-O-Glucoside* | Flavonoids | Flavonols | 614000 | 668000 | 969000 | 789000 | 913000 | 841000 | 481000 | 625000 | 484000 | 894000 | 1070000 | 1880000 | 574000 | 604000 | 544000 |
| Myricetin-3-O-glucoside* | Flavonoids | Flavonols | 9 | 9 | 9 | 9 | 9 | 9 | 60900 | 63000 | 58500 | 29200 | 36600 | 17700 | 239000 | 274000 | 260000 |
| Kaempferol-3-O-(6''-acetyl) glucoside | Flavonoids | Flavonols | 26600 | 21000 | 38900 | 68700 | 84700 | 65800 | 21300 | 16000 | 14200 | 219000 | 180000 | 396000 | 18600 | 13400 | 15400 |
| Kaempferol-3-O-(2''-acetyl) glucoside | Flavonoids | Flavonols | 8690 | 6240 | 9890 | 48000 | 26800 | 48100 | 10600 | 17100 | 19600 | 17400 | 20400 | 26800 | 22700 | 28900 | 22800 |
| Quercetin-3-O-(6''-acetyl) galactoside | Flavonoids | Flavonols | 157000 | 75300 | 147000 | 254000 | 250000 | 242000 | 98800 | 81600 | 108000 | 273000 | 669000 | 426000 | 13200 | 13100 | 14300 |
| Kaempferol-3-O-(6''-malonyl) galactoside* | Flavonoids | Flavonols | 28700 | 18500 | 21100 | 1220000 | 1320000 | 1080000 | 106000 | 84900 | 91600 | 559000 | 705000 | 797000 | 61100 | 65900 | 53900 |
| Kaempferol-3-O-(6''-malonyl) glucoside* | Flavonoids | Flavonols | 70700 | 75500 | 95800 | 514000 | 526000 | 522000 | 84600 | 78600 | 76300 | 578000 | 862000 | 1110000 | 60700 | 60500 | 63300 |
| Quercetin-3-O-(6''-malonyl) galactoside* | Flavonoids | Flavonols | 309000 | 305000 | 401000 | 623000 | 769000 | 655000 | 257000 | 262000 | 227000 | 1190000 | 1370000 | 1740000 | 38400 | 37500 | 32700 |
| Quercetin-7-O-(6''-malonyl) glucoside* | Flavonoids | Flavonols | 291000 | 336000 | 492000 | 543000 | 604000 | 563000 | 276000 | 262000 | 252000 | 989000 | 1020000 | 1380000 | 39300 | 42700 | 30700 |
| Kaempferol-3,7-O-dirhamnoside (Kaempferitrin)* | Flavonoids | Flavonols | 12800000 | 12700000 | 11900000 | 4700000 | 5150000 | 4770000 | 3540000 | 2710000 | 3490000 | 1160000 | 1480000 | 1240000 | 2150000 | 2940000 | 2550000 |
| Kaempferol-3-O-(6''-p-coumaroyl) glucoside (Tiliroside)* | Flavonoids | Flavonols | 9 | 9 | 9 | 1490000 | 1480000 | 1360000 | 1790000 | 2230000 | 1790000 | 3520000 | 3970000 | 3700000 | 2270000 | 2070000 | 2300000 |
| Kaempferol-3-O-neohesperidoside | Flavonoids | Flavonols | 79200 | 44600 | 26800 | 5130000 | 5660000 | 5000000 | 8100000 | 9220000 | 8020000 | 11000000 | 12500000 | 10100000 | 7860000 | 7900000 | 8290000 |
| Quercetin-3-O-apiosyl (1→2) galactoside* | Flavonoids | Flavonols | 194000 | 244000 | 357000 | 280000 | 252000 | 314000 | 269000 | 227000 | 240000 | 352000 | 439000 | 572000 | 127000 | 120000 | 135000 |
| Quercetin-3-O-xylosyl (1→2) glucoside* | Flavonoids | Flavonols | 228000 | 230000 | 369000 | 272000 | 265000 | 284000 | 293000 | 282000 | 254000 | 372000 | 401000 | 581000 | 138000 | 141000 | 129000 |
| Quercetin-3-O-(6''-p-Coumaroyl) galactoside | Flavonoids | Flavonols | 238000 | 339000 | 589000 | 772000 | 947000 | 932000 | 305000 | 423000 | 216000 | 109000 | 220000 | 325000 | 9 | 9 | 9 |
| Quercetin-3-O-neohesperidoside* | Flavonoids | Flavonols | 436000 | 571000 | 775000 | 598000 | 761000 | 539000 | 365000 | 372000 | 401000 | 765000 | 707000 | 992000 | 451000 | 900000 | 730000 |
| 6-C-Methylquercetin-3-O-rutinoside | Flavonoids | Flavonols | 9 | 9 | 9 | 51900 | 14900 | 147000 | 9 | 9 | 9 | 63900 | 297000 | 64400 | 7710 | 12200 | 8440 |
| 6-Hydroxykaempferol-7,6-O-Diglucoside | Flavonoids | Flavonols | 128000 | 138000 | 283000 | 39500 | 42900 | 49700 | 44700 | 56200 | 58400 | 67200 | 130000 | 178000 | 18500 | 21300 | 20600 |
| Quercetin-3-O-(2''-O-arabinosyl) rutinoside | Flavonoids | Flavonols | 14000000 | 15400000 | 19300000 | 13700000 | 13700000 | 12300000 | 6710000 | 7280000 | 7080000 | 17500000 | 21300000 | 21600000 | 22700000 | 22500000 | 22000000 |
| Kaempferol-3-O-rutinoside-7-O-glucoside | Flavonoids | Flavonols | 417000 | 287000 | 316000 | 14000000 | 11700000 | 12700000 | 1390000 | 975000 | 1150000 | 2780000 | 2620000 | 2550000 | 1750000 | 1570000 | 1370000 |
| Quercetin-3-O-rutinoside-7-O-rhamnoside* | Flavonoids | Flavonols | 57700 | 44300 | 41100 | 1960000 | 1730000 | 1730000 | 146000 | 90300 | 108000 | 259000 | 249000 | 270000 | 144000 | 242000 | 179000 |
| Quercetin-7-O-rutinoside-4'-O-glucoside | Flavonoids | Flavonols | 754000 | 772000 | 888000 | 989000 | 872000 | 871000 | 625000 | 740000 | 598000 | 884000 | 1100000 | 1010000 | 471000 | 545000 | 459000 |
| Quercetin-3-O-rutinoside-7-O-glucoside* | Flavonoids | Flavonols | 71100 | 45800 | 68500 | 59100 | 66700 | 65200 | 113000 | 95400 | 110000 | 69600 | 91000 | 71500 | 81100 | 85200 | 61200 |
| 2'-Hydroxyisoflavone | Flavonoids | Isoflavones | 2450 | 2250 | 1440 | 2940 | 3330 | 2680 | 1220 | 1630 | 1620 | 3070 | 4770 | 8220 | 44400 | 44000 | 41000 |
| Irilin D | Flavonoids | Isoflavones | 9 | 9 | 9 | 2640 | 1210 | 2180 | 9 | 9 | 9 | 1610 | 9850 | 3610 | 1220 | 2250 | 942 |
| Genistein-8-C-glucoside* | Flavonoids | Isoflavones | 538000 | 717000 | 749000 | 362000 | 501000 | 347000 | 59900 | 43700 | 93400 | 158000 | 329000 | 53800 | 617000 | 796000 | 633000 |
| 5,7,4'-Trihydroxyisoflavone-7-O-galactoside* | Flavonoids | Isoflavones | 1910000 | 2030000 | 1690000 | 271000 | 283000 | 357000 | 1700000 | 1620000 | 2220000 | 834000 | 1170000 | 661000 | 19000000 | 19500000 | 15800000 |
| 6,4'-Dimethoxyisoflavone-7-O-glucoside (Wistin) | Flavonoids | Isoflavones | 23000 | 26100 | 13200 | 19700 | 19100 | 19900 | 92500 | 87000 | 94800 | 46800 | 48900 | 33000 | 246000 | 266000 | 226000 |
| 5,7,4'-Trihydroxyisoflavone-7-O-galactoside-rhamnose* | Flavonoids | Isoflavones | 2330000 | 2150000 | 2000000 | 3950000 | 3020000 | 3610000 | 3430000 | 3550000 | 3310000 | 7580000 | 7870000 | 6570000 | 7630000 | 5790000 | 6270000 |
| 2'-Hydoxy,5-methoxyGenistein-4',7-O-diglucoside | Flavonoids | Isoflavones | 9 | 9 | 17100 | 270000 | 115000 | 357000 | 9 | 6060 | 9 | 71500 | 194000 | 179000 | 40900 | 171000 | 124000 |
| Theaflavin | Tannins | Proanthocyanidins | 2210 | 1910 | 1620 | 1350 | 1540 | 1300 | 2310 | 2710 | 4950 | 1080 | 1810 | 904 | 2030 | 3690 | 1790 |
| 2α,3α-Epoxy-5,7,3',4'-tetrahydroxyflavan-(4β-8-catechin)* | Tannins | Proanthocyanidins | 645000 | 474000 | 192000 | 714000 | 906000 | 1050000 | 203000 | 181000 | 244000 | 471000 | 330000 | 581000 | 381000 | 239000 | 272000 |
| Procyanidin A1 | Tannins | Proanthocyanidins | 94600 | 105000 | 127000 | 145000 | 124000 | 176000 | 76200 | 59600 | 88700 | 66300 | 70800 | 58700 | 93600 | 123000 | 95600 |
| Procyanidin A2* | Tannins | Proanthocyanidins | 201000 | 263000 | 323000 | 364000 | 307000 | 360000 | 199000 | 129000 | 204000 | 186000 | 190000 | 130000 | 200000 | 274000 | 249000 |
| Procyanidin B1 | Tannins | Proanthocyanidins | 18100000 | 19600000 | 21700000 | 26100000 | 27600000 | 27400000 | 20400000 | 20500000 | 19100000 | 19100000 | 21200000 | 22800000 | 11200000 | 14200000 | 11800000 |
| Procyanidin B2 | Tannins | Proanthocyanidins | 6040000 | 7170000 | 4340000 | 7850000 | 7670000 | 8060000 | 6620000 | 6750000 | 7510000 | 5950000 | 7730000 | 8430000 | 9650000 | 10700000 | 11600000 |
| Procyanidin B3 | Tannins | Proanthocyanidins | 5600000 | 7010000 | 4270000 | 7560000 | 8320000 | 8430000 | 7200000 | 6500000 | 7150000 | 5660000 | 8340000 | 8240000 | 9810000 | 11700000 | 11200000 |
| Procyanidin B4 | Tannins | Proanthocyanidins | 23700000 | 32200000 | 28900000 | 20500000 | 19800000 | 22900000 | 19900000 | 18100000 | 19700000 | 10700000 | 11500000 | 10300000 | 15400000 | 16300000 | 17200000 |
| Procyanidin A6 | Tannins | Proanthocyanidins | 347000 | 522000 | 345000 | 428000 | 444000 | 401000 | 591000 | 482000 | 476000 | 207000 | 297000 | 261000 | 349000 | 408000 | 351000 |
| (EC→EC) g(1) | Tannins | Proanthocyanidins | 1960000 | 2020000 | 1970000 | 1400000 | 1470000 | 1830000 | 396000 | 686000 | 559000 | 247000 | 514000 | 375000 | 1180000 | 1800000 | 1340000 |
| (EC→EC→EC) g(5) | Tannins | Proanthocyanidins | 44500 | 37400 | 43500 | 37700 | 50900 | 37600 | 7400 | 11000 | 20200 | 4080 | 9130 | 6020 | 21100 | 22400 | 21400 |
| (EC→EC→EC) g(1) | Tannins | Proanthocyanidins | 22200 | 18500 | 18600 | 20400 | 27100 | 18100 | 8440 | 10500 | 11300 | 9670 | 8970 | 5310 | 15700 | 18900 | 12800 |
| (EC→EC→EC) g(2) | Tannins | Proanthocyanidins | 62700 | 53800 | 77000 | 52300 | 62000 | 64400 | 12700 | 19400 | 19800 | 11500 | 14000 | 12600 | 26600 | 59700 | 41600 |
| (EC→EC→EC) g(4) | Tannins | Proanthocyanidins | 55100 | 65300 | 64700 | 29800 | 34300 | 40800 | 7210 | 6590 | 12700 | 6810 | 7990 | 5590 | 25100 | 41800 | 26000 |
| (EC→EC→EC) g(6) | Tannins | Proanthocyanidins | 36700 | 33800 | 45200 | 38600 | 37800 | 43200 | 11300 | 6540 | 18100 | 8780 | 8180 | 6800 | 20300 | 22900 | 19000 |
| Procyanidin C1 3'-O-gallate | Tannins | Proanthocyanidins | 128000 | 132000 | 221000 | 52300 | 39600 | 51300 | 7990 | 14800 | 14300 | 9 | 9 | 9 | 16200 | 47300 | 28100 |
| Gallic acid | Tannins | Tannin | 15000000 | 16900000 | 1440000 | 1030000 | 1010000 | 1050000 | 163000 | 271000 | 628000 | 3160000 | 4590000 | 3740000 | 6430000 | 7140000 | 7270000 |
| 3-O-Methylgallic Acid | Tannins | Tannin | 50200 | 53200 | 18900 | 12700 | 15300 | 16900 | 3900 | 4200 | 11200 | 9950 | 16700 | 9500 | 19600 | 21500 | 20400 |
| Gallic acid-4-O-glucoside | Tannins | Tannin | 111000 | 92600 | 72400 | 74000 | 143000 | 78900 | 22900 | 12900 | 27500 | 60300 | 37500 | 154000 | 30800 | 33500 | 30700 |
| 6-O-Galloyl-glucose | Tannins | Tannin | 38300000 | 47900000 | 7650000 | 5170000 | 5400000 | 4680000 | 1900000 | 2630000 | 5210000 | 5460000 | 7030000 | 6730000 | 21600000 | 27000000 | 25600000 |
| Theaflagallin | Tannins | Tannin | 11400 | 11400 | 6810 | 7910 | 7780 | 4970 | 7030 | 7310 | 7780 | 5580 | 4860 | 6450 | 6310 | 5730 | 3850 |
| Theaflavic acid | Tannins | Tannin | 48000 | 117000 | 43300 | 88000 | 46000 | 92500 | 88700 | 123000 | 78100 | 23900 | 28200 | 73200 | 75300 | 38800 | 70700 |
| Ellagic acid-4-O-glucoside | Tannins | Tannin | 25900 | 20800 | 19300 | 54600 | 68800 | 68700 | 9 | 9 | 9 | 38000 | 78400 | 9 | 9 | 9 | 9 |
| 1,4-Di-O-galloyl-glcose | Tannins | Tannin | 2540000 | 4740000 | 431000 | 277000 | 309000 | 281000 | 188000 | 235000 | 415000 | 585000 | 1470000 | 158000 | 1020000 | 1170000 | 1170000 |
| Digalloylglucose* | Tannins | Tannin | 527000 | 602000 | 82200 | 63900 | 68000 | 62600 | 6950 | 9520 | 38100 | 51400 | 63400 | 69700 | 267000 | 225000 | 212000 |
| 2,3-Di-O-Galloyl-D-Glucose* | Tannins | Tannin | 737000 | 749000 | 107000 | 82700 | 93500 | 75600 | 8430 | 11600 | 51400 | 65900 | 83800 | 88900 | 314000 | 286000 | 253000 |
| Epitheaflavic acid-3-O-Gallate | Tannins | Tannin | 7420000 | 7610000 | 7580000 | 7000000 | 7710000 | 7310000 | 7650000 | 7750000 | 7320000 | 4940000 | 5920000 | 5600000 | 6930000 | 7490000 | 7090000 |
| Gambiriin A1 | Tannins | Tannin | 1140000 | 1100000 | 1270000 | 1190000 | 982000 | 1210000 | 1300000 | 1040000 | 1160000 | 657000 | 905000 | 889000 | 1020000 | 1240000 | 1160000 |
| Maplexin H | Tannins | Tannin | 232000 | 363000 | 39400 | 41100 | 33000 | 55000 | 12400 | 15600 | 22000 | 23500 | 65100 | 27400 | 88600 | 95200 | 87800 |
| Gemin D | Tannins | Tannin | 23000 | 28400 | 16000 | 20100 | 18200 | 21300 | 26200 | 29000 | 31200 | 13600 | 8490 | 21800 | 16800 | 18600 | 16200 |
| Maplexin G | Tannins | Tannin | 4760 | 4350 | 11600 | 14000 | 16000 | 13500 | 2670 | 2110 | 6230 | 5220 | 9330 | 2270 | 11500 | 15600 | 9860 |
| 1,3,6-Tri-O-galloyl-D-glucose | Tannins | Tannin | 391000 | 376000 | 44700 | 50900 | 59300 | 51600 | 14400 | 15200 | 36200 | 32300 | 107000 | 34900 | 186000 | 227000 | 222000 |
| 2,4,6-Tri-O-galloyl-D-glucose | Tannins | Tannin | 14700 | 18100 | 4350 | 7120 | 8320 | 7980 | 2380 | 2230 | 2690 | 49400 | 58400 | 4820 | 2100 | 4840 | 1350 |
| Digalloyl-HHDP-glucose | Tannins | Tannin | 15400 | 17900 | 3580 | 10600 | 8680 | 12000 | 1280 | 1540 | 2590 | 73100 | 251000 | 2170 | 31000 | 32600 | 19500 |
| Cinnamtannin B1* | Tannins | Tannin | 2020000 | 1720000 | 1020000 | 2730000 | 3130000 | 2400000 | 1400000 | 1300000 | 1530000 | 2350000 | 2270000 | 4120000 | 1990000 | 1800000 | 1720000 |
| Cinnamtannin D1* | Tannins | Tannin | 460000 | 429000 | 407000 | 604000 | 518000 | 552000 | 308000 | 295000 | 281000 | 456000 | 382000 | 487000 | 225000 | 236000 | 197000 |
| Arecatannin A2 | Tannins | Tannin | 1070000 | 1500000 | 1530000 | 1180000 | 1330000 | 1180000 | 1410000 | 1240000 | 1310000 | 998000 | 1120000 | 1090000 | 678000 | 772000 | 790000 |
| Cinnamtannin A2 | Tannins | Tannin | 1420000 | 1310000 | 1320000 | 752000 | 1240000 | 978000 | 993000 | 1190000 | 981000 | 624000 | 985000 | 849000 | 831000 | 794000 | 842000 |
| Cinnamtannin B2 | Tannins | Tannin | 1140000 | 903000 | 1140000 | 784000 | 937000 | 1020000 | 1530000 | 1370000 | 1170000 | 1000000 | 1100000 | 1340000 | 887000 | 763000 | 930000 |

**Supplementary Table S3. Summary of sequencing data.**

| Sample | RawData | CleanData (%) | Adapter (%) | LowQuality (%) | polyA (%) | N (%) |
| --- | --- | --- | --- | --- | --- | --- |
| CK1 | 58,818,496 | 58,679,516 (99.76%) | 22,056 (0.04%) | 116,924 (0.20%) | 0 (0.00%) | 0 (0.00%) |
| CK2 | 45,466,448 | 45,354,024 (99.75%) | 19,286 (0.04%) | 93,138 (0.20%) | 0 (0.00%) | 0 (0.00%) |
| CK3 | 52,586,430 | 52,499,070 (99.83%) | 13,810 (0.03%) | 73,550 (0.14%) | 0 (0.00%) | 0 (0.00%) |
| T1-1 | 39,418,728 | 39,308,166 (99.72%) | 23,676 (0.06%) | 86,886 (0.22%) | 0 (0.00%) | 0 (0.00%) |
| T1-2 | 51,461,748 | 51,333,522 (99.75%) | 20,972 (0.04%) | 107,254 (0.21%) | 0 (0.00%) | 0 (0.00%) |
| T1-3 | 51,442,558 | 51,318,532 (99.76%) | 28,588 (0.06%) | 95,438 (0.19%) | 0 (0.00%) | 0 (0.00%) |
| T2-1 | 49,285,938 | 49,180,808 (99.79%) | 18,076 (0.04%) | 87,054 (0.18%) | 0 (0.00%) | 0 (0.00%) |
| T2-2 | 49,389,354 | 49,281,678 (99.78%) | 20,886 (0.04%) | 86,790 (0.18%) | 0 (0.00%) | 0 (0.00%) |
| T2-3 | 52,425,276 | 52,317,576 (99.79%) | 19,186 (0.04%) | 88,514 (0.17%) | 0 (0.00%) | 0 (0.00%) |
| T3-1 | 47,572,158 | 47,473,898 (99.79%) | 18,244 (0.04%) | 80,014 (0.17%) | 0 (0.00%) | 2 (0.00%) |
| T3-2 | 42,332,130 | 42,242,210 (99.79%) | 14,626 (0.03%) | 75,294 (0.18%) | 0 (0.00%) | 0 (0.00%) |
| T3-3 | 52,755,756 | 52,627,694 (99.76%) | 23,202 (0.04%) | 104,860 (0.20%) | 0 (0.00%) | 0 (0.00%) |
| T4-1 | 50,112,092 | 49,981,452 (99.74%) | 23,894 (0.05%) | 106,746 (0.21%) | 0 (0.00%) | 0 (0.00%) |
| T4-2 | 39,188,334 | 39,093,790 (99.76%) | 17,184 (0.04%) | 77,360 (0.20%) | 0 (0.00%) | 0 (0.00%) |
| T4-3 | 56,169,142 | 56,044,684 (99.78%) | 22,128 (0.04%) | 102,330 (0.18%) | 0 (0.00%) | 0 (0.00%) |

**Supplementary Table S4. Quality inspection of sample sequencing data.**

| **Sample** | **RawData (bp)** | **CleanData (bp)** | **Q20 (%)** | **Q30 (%)** | **N (%)** | **GC (%)** |
| --- | --- | --- | --- | --- | --- | --- |
| CK1 | 8,822,774,400 | 8,730,082,023 | 8,458,584,794 (96.89%) | 8,014,157,463 (91.80%) | 17,573 (0.00%) | 4,063,481,007 (46.55%) |
| CK2 | 6,819,967,200 | 6,747,121,468 | 6,543,777,248 (96.99%) | 6,205,615,650 (91.97%) | 13,640 (0.00%) | 3,128,906,138 (46.37%) |
| CK3 | 7,887,964,500 | 7,798,418,749 | 7,591,215,219 (97.34%) | 7,224,442,088 (92.64%) | 15,572 (0.00%) | 3,680,539,496 (47.20%) |
| T1-1 | 5,912,809,200 | 5,836,869,376 | 5,654,805,214 (96.88%) | 5,358,611,831 (91.81%) | 11,917 (0.00%) | 2,700,838,273 (46.27%) |
| T1-2 | 7,719,262,200 | 7,629,746,827 | 7,390,547,241 (96.86%) | 7,003,332,649 (91.79%) | 15,241 (0.00%) | 3,525,788,443 (46.21%) |
| T1-3 | 7,716,383,700 | 7,621,251,723 | 7,411,335,625 (97.25%) | 7,055,805,568 (92.58%) | 14,292 (0.00%) | 3,559,105,781 (46.70%) |
| T2-1 | 7,392,890,700 | 7,312,955,225 | 7,093,971,157 (97.01%) | 6,729,933,560 (92.03%) | 14,719 (0.00%) | 3,395,959,357 (46.44%) |
| T2-2 | 7,408,403,100 | 7,335,553,489 | 7,131,496,175 (97.22%) | 6,783,086,215 (92.47%) | 14,146 (0.00%) | 3,406,465,933 (46.44%) |
| T2-3 | 7,863,791,400 | 7,781,379,328 | 7,568,030,148 (97.26%) | 7,201,243,676 (92.54%) | 15,848 (0.00%) | 3,637,785,558 (46.75%) |
| T3-1 | 7,135,823,700 | 7,068,668,336 | 6,876,131,164 (97.28%) | 6,546,603,886 (92.61%) | 13,527 (0.00%) | 3,279,603,760 (46.40%) |
| T3-2 | 6,349,819,500 | 6,280,864,725 | 6,099,963,446 (97.12%) | 5,794,859,707 (92.26%) | 12,635 (0.00%) | 2,928,844,350 (46.63%) |
| T3-3 | 7,913,363,400 | 7,809,934,695 | 7,573,166,270 (96.97%) | 7,186,005,737 (92.01%) | 14,831 (0.00%) | 3,630,447,474 (46.48%) |
| T4-1 | 7,516,813,800 | 7,422,662,534 | 7,179,044,652 (96.72%) | 6,786,882,023 (91.43%) | 15,011 (0.00%) | 3,542,719,599 (47.73%) |
| T4-2 | 5,878,250,100 | 5,803,770,479 | 5,639,726,394 (97.17%) | 5,361,817,259 (92.39%) | 11,796 (0.00%) | 2,709,684,407 (46.69%) |
| T4-3 | 8,425,371,300 | 8,354,781,001 | 8,112,875,077 (97.10%) | 7,699,694,527 (92.16%) | 16,929 (0.00%) | 3,960,039,413 (47.40%) |

**Supplementary Table S5. Detection of sample alignment rate.**

| **Sample** | **Total** | **Unmapped (%)** | **Unique_Mapped (%)** | **Multiple_Mapped (%)** | **Total_Mapped (%)** |
| --- | --- | --- | --- | --- | --- |
| CK1 | 58,327,982 | 13,644,258 (23.39%) | 37,474,165 (64.25%) | 7,209,559 (12.36%) | 44,683,724 (76.61%) |
| CK2 | 44,760,668 | 10,486,416 (23.43%) | 28,701,983 (64.12%) | 5,572,269 (12.45%) | 34,274,252 (76.57%) |
| CK3 | 51,895,842 | 11,995,082 (23.11%) | 33,623,182 (64.79%) | 6,277,578 (12.10%) | 39,900,760 (76.89%) |
| T1-1 | 38,964,936 | 9,823,283 (25.21%) | 24,410,079 (62.65%) | 4,731,574 (12.14%) | 29,141,653 (74.79%) |
| T1-2 | 50,842,390 | 12,662,042 (24.90%) | 31,841,798 (62.63%) | 6,338,550 (12.47%) | 38,180,348 (75.10%) |
| T1-3 | 50,229,472 | 12,321,950 (24.53%) | 31,624,838 (62.96%) | 6,282,684 (12.51%) | 37,907,522 (75.47%) |
| T2-1 | 47,799,178 | 12,712,761 (26.60%) | 29,306,772 (61.31%) | 5,779,645 (12.09%) | 35,086,417 (73.40%) |
| T2-2 | 48,051,124 | 12,187,826 (25.36%) | 30,041,012 (62.52%) | 5,822,286 (12.12%) | 35,863,298 (74.64%) |
| T2-3 | 50,744,390 | 12,718,628 (25.06%) | 31,916,308 (62.90%) | 6,109,454 (12.04%) | 38,025,762 (74.94%) |
| T3-1 | 46,952,294 | 10,907,409 (23.23%) | 30,033,270 (63.97%) | 6,011,615 (12.80%) | 36,044,885 (76.77%) |
| T3-2 | 41,636,962 | 9,701,334 (23.30%) | 26,822,745 (64.42%) | 5,112,883 (12.28%) | 31,935,628 (76.70%) |
| T3-3 | 52,133,058 | 12,395,466 (23.78%) | 32,892,789 (63.09%) | 6,844,803 (13.13%) | 39,737,592 (76.22%) |
| T4-1 | 45,019,216 | 11,566,566 (25.69%) | 28,379,838 (63.04%) | 5,072,812 (11.27%) | 33,452,650 (74.31%) |
| T4-2 | 38,751,246 | 9,894,031 (25.53%) | 24,476,927 (63.16%) | 4,380,288 (11.30%) | 28,857,215 (74.47%) |
| T4-3 | 53,069,680 | 13,262,294 (24.99%) | 33,875,802 (63.83%) | 5,931,584 (11.18%) | 39,807,386 (75.01%) |

**Supplementary Table S6. Assessment of sample alignment area.**

| **Sample** | **Exon** | **Intron** | **Intergenic** |
| --- | --- | --- | --- |
| CK1 | 39,702,538 (88.85%) | 1,103,254 (2.47%) | 3,877,932 (8.68%) |
| CK2 | 30,408,068 (88.72%) | 8,820,80 (2.57%) | 2,984,104 (8.71%) |
| CK3 | 35,133,382 (88.05%) | 1,082,458 (2.71%) | 3,684,920 (9.24%) |
| T1-1 | 25,512,431 (87.55%) | 922,246 (3.16%) | 2,706,976 (9.29%) |
| T1-2 | 33,602,774 (88.01%) | 1,178,666 (3.09%) | 3,398,908 (8.90%) |
| T1-3 | 33,280,925 (87.80%) | 1,157,849 (3.05%) | 3,468,748 (9.15%) |
| T2-1 | 30,783,607 (87.74%) | 1,062,626 (3.03%) | 3,240,184 (9.23%) |
| T2-2 | 31,504,130 (87.85%) | 1,026,653 (2.86%) | 3,332,515 (9.29%) |
| T2-3 | 33,515,892 (88.14%) | 1,024,860 (2.70%) | 3,485,010 (9.16%) |
| T3-1 | 31,630,450 (87.75%) | 1,104,834 (3.07%) | 3,309,601 (9.18%) |
| T3-2 | 28,040,129 (87.80%) | 932,685 (2.92%) | 2,962,814 (9.28%) |
| T3-3 | 35,098,815 (88.33%) | 1,152,320 (2.90%) | 3,486,457 (8.77%) |
| T4-1 | 29,116,623 (87.04%) | 1,007,173 (3.01%) | 3,328,854 (9.95%) |
| T4-2 | 25,039,428 (86.77%) | 927,262 (3.21%) | 2,890,525 (10.02%) |
| T4-3 | 34,692,320 (87.15%) | 1,187,936 (2.98%) | 3,927,130 (9.87%) |

**Supplementary Figure S1. Sample correlation analysis and comparative analysis of DEGs.**

**Supplementary Figure S2. The GO and KEGG enrichment results of overlapping DEGs (634 DEGs).**

**Supplementary** **Figure S3**. The identification results of TFs related to anthocyanin accumulation.

**Supplementary** **Figure S4**. The expression level of DEGs in different modules.

**Supplementary** **Figure S5**. qRT-PCR validation of gene expression level in the transcriptome.

**Supplementary** **Figure S6**. Correlation analysis of the results between RNA-seq and qRT-PCR.
